# Pentacyclic Triterpenoid Acids in the Treatment of Breast Cancer

**DOI:** 10.3390/molecules31183207

**Published:** 2026-09-11

**Authors:** Natalia Jankowska, Rostyslav Dudchak, Magdalena Podolak, Marcin Cybulski, Elisabetta Esposito, Olga Michalak, Krzysztof Bielawski, Anna Bielawska, Agnieszka Gornowicz

**Affiliations:** 1 Department of Biotechnology, Medical University of Bialystok, 1 Kilińskiego Str., 15-089 Białystok, Poland; magdalena.podolak@sd.umb.edu.pl (M.P.); anna.bielawska@umb.edu.pl (A.B.); 2 Department of Synthesis and Technology of Drugs, Medical University of Bialystok, 1 Kilińskiego Str., 15-089 Białystok, Poland; rostyadudchak@gmail.com (R.D.); krzysztof.bielawski@umb.edu.pl (K.B.); 3 Chemistry Section, Pharmacy, Cosmetic Chemistry and Biotechnology Research Group, Łukasiewicz Research Network–Industrial Chemistry Institute, 8 Rydygiera Str., 01-793 Warsaw, Poland; marcin.cybulski@ichp.lukasiewicz.gov.pl (M.C.); olga.michalak@ichp.lukasiewicz.gov.pl (O.M.); 4 Department of Chemical, Pharmaceutical and Agricultural Sciences, University of Ferrara, Via Fossato di Mortara, 19-44121 Ferrara, Italy; ese@unife.it

**Keywords:** pentacyclic triterpenoids, anticancer, breast cancer

## Abstract

Despite increasing awareness about cancer, it still remains one of the biggest threats to human life. Breast cancer is among the most commonly diagnosed types of cancer. Pentacyclic triterpenoids are a group of plant compounds commonly known in traditional medicine due to their anti-inflammatory, antibacterial, antidiabetic, cardioprotective and anticancer effects. This review examined the potential of pentacyclic triterpenoid acids in the treatment of breast cancer, focusing on research from the past 15 years. Although the subject literature demonstrates that pentacyclic acids show anticancer activity, it also reveals their limitations such as low bioavailability, aqueous solubility, or high IC_50_ values. Therefore, to overcome these limitations, structural modifications, mostly at the C3 and C28 positions, were made, and several novel derivatives with oleanane, lupane and ursane skeletal types were synthesized. This review not only identified pentacyclic acids derivatives but also evaluated how structural modifications enhanced their biological activity and potential in breast cancer treatment. The findings revealed that even though the obtained derivatives enhanced anticancer potential against breast cancer, the main issues of bioavailability and solubility remain unsolved, suggesting that future research in synthesis should focus not only on improving cytotoxicity but also on the bioavailability of novel compounds.

## 1. Introduction

Cancer remains one of the most important challenges in the field of medicinal chemistry. Despite the notable advancements made by scientists in researching the possible treatments for this disease, the ultimate solution has yet to be found. Breast cancer is of particular interest as it is the most commonly diagnosed type of cancer worldwide, with a continuously rising trend of approximately 1% every year in the years 2012–2021. For instance, in 2020, 2.3 million new cases of breast cancer were diagnosed, and 685,000 deaths were noted [1]. Such threatening statistics become especially dangerous considering future predictions. According to Arnold et al., the number of new and fatal cases will rise to 3 million new cases and 1 million deaths, respectively, by 2040 [1,2]. Cases of drug-resistant breast cancer are particularly challenging, since typically applied chemotherapy with currently existing drugs may not provide positive results, leaving the patient without treatment options. Particularly known types are triple-negative and HER2 positive breast cancers, which cause hormone and endocrine instability. Other factors, such as resistant proteins and p-gp efflux of drugs, also contribute to such resistance [3]. Given these factors, the development of new potential therapeutics for breast cancer has become a key area of focus in the current field of medicinal chemistry.

**Figure 1:**
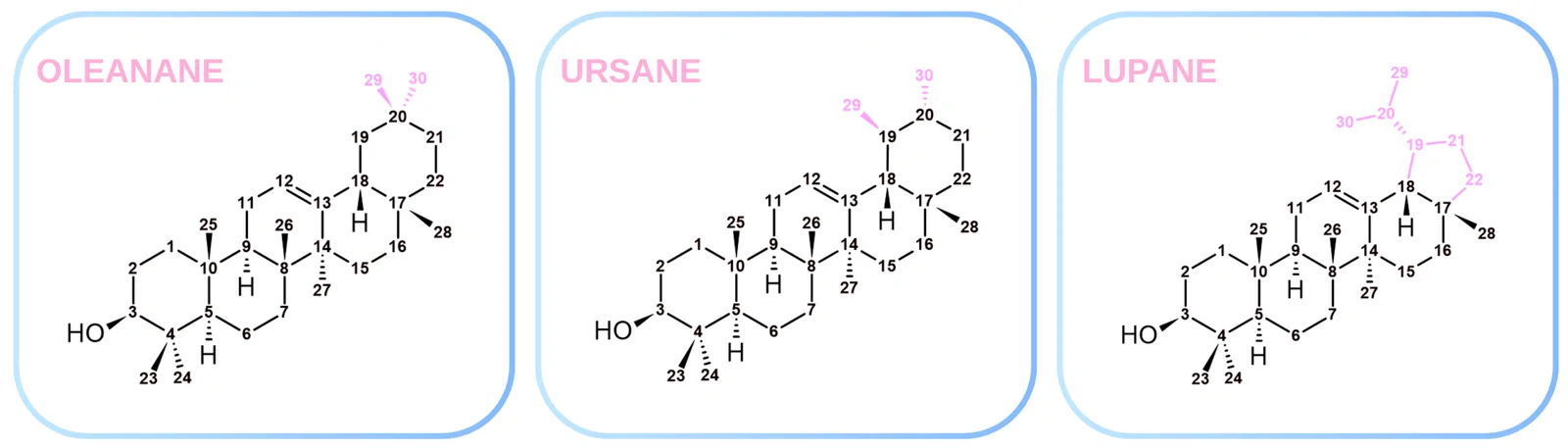
Structural frameworks of the oleanane, ursane, and lupane pentacyclic triterpenoid skeletal types. The structural differences between skeletal types are highlighted in pink. Numbers correspond to the locations of carbon atoms of respective structures.

More than 60% of currently used anticancer drugs are based on structures found in the natural environment [4]. Hence, scientists are constantly searching, verifying and modifying plant-derived compounds for their anticancer properties. Pentacyclic triterpenoid acids are notably interesting, being a common group in the medicinal plant world. Long recognized in traditional medicine, they have been widely used for a variety of ailments. They are primarily known for their anti-inflammatory, antioxidant, antibacterial, and antiviral properties [5,6,7]. Additionally, over the years and through research, their antidiabetic, hepatoprotective, cardioprotective, and anticancer properties have also been discovered [8,9,10]. They are all characterized by possessing a 30-carbon skeleton with five interconnected rings, creating a complex, highly stable structure with high melting points and significant hydrophobic characteristics [11]. Depending on their molecular structure, pentacyclic acids can be divided into three skeletal types: lupane, oleanane, and ursane (Figure 1) [12]. Nowadays, the relevant acids are widely distributed in dietary supplements, supporting joints and bones, skin conditions, and cognitive functions, also acting as cytoprotective and antioxidant preparations [12]. Despite the wide use of pentacyclic triterpenoids, only one semi-synthetic oleanolic acid derivative, omaveloxolone, was approved by the Food and Drug Administration (FDA) in 2023 as the first and only drug for the treatment of Friedreich’s ataxia [13]. In this paper, we aim to thoroughly analyze the anticancer properties of pentacyclic triterpenoid acids and their derivatives against breast cancer over the past 15 years.

## 2. Oleanane Skeletal Type

**Figure 2:**
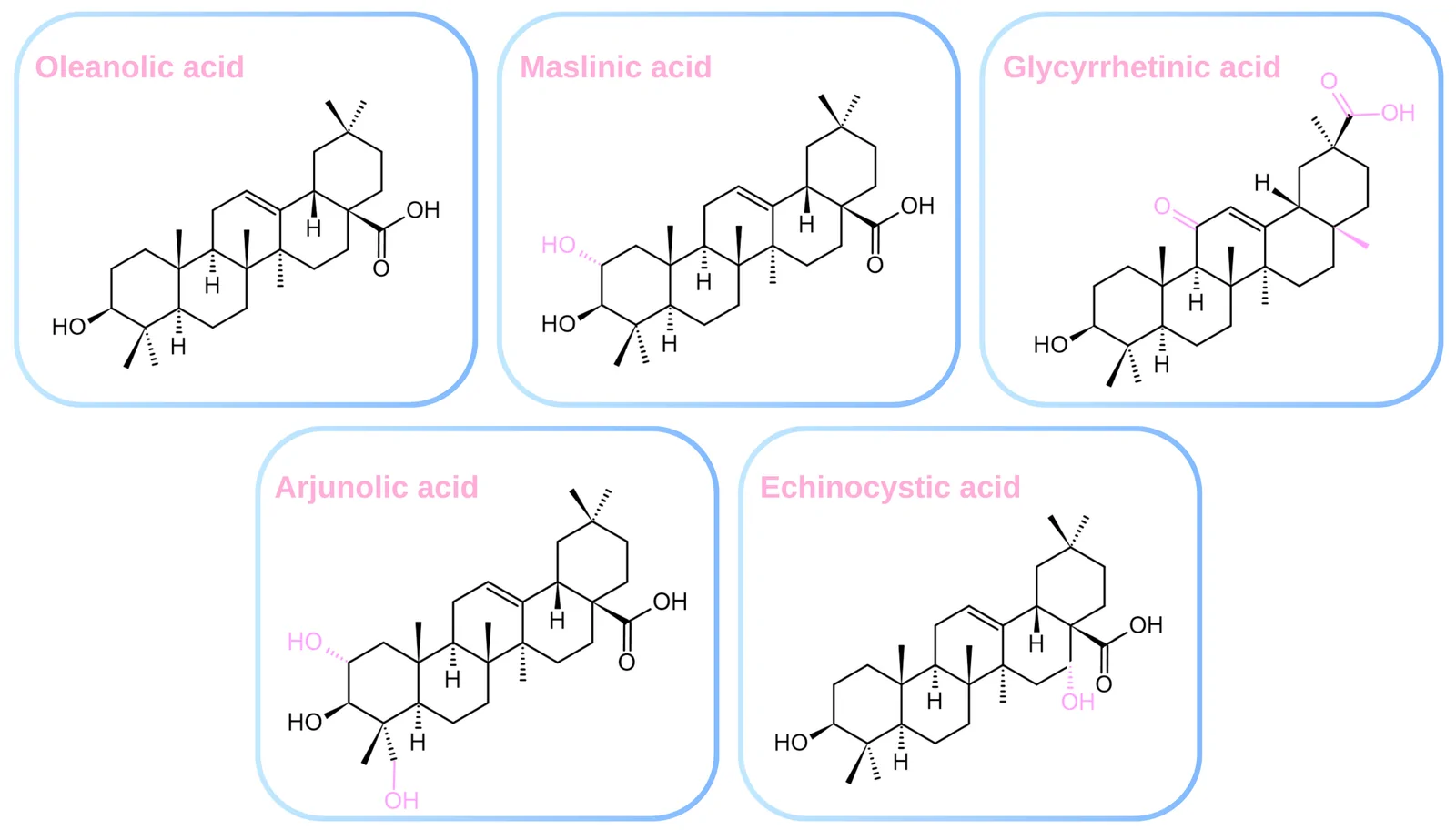
The structures of natural pentacyclic triterpenoid acids with oleanane skeletal type. The structural differences between the compounds are highlighted in pink.

Among pentacyclic triterpenoid acids, compounds with the oleanane skeleton are the most commonly researched. Oleanane’s main structural difference from other skeletal types lies in the gem-dimethyl group (C29 and C30) connected to one atom of the E-ring (C20). Five pentacyclic triterpenoid acids with this skeletal type and their derivatives were analyzed in this review: oleanolic, maslinic, glycyrrhetinic, arjunolic and echinocystic (Figure 2). All of them feature at least one hydroxyl group at the C3 position and carboxylic group, which can be connected to either C17, as shown in oleanolic, maslinic, arjunolic and echinocystic acids, or the C20 atom, as visible in glycyrrhetinic acid, of the E-ring of oleanane core. Another notable difference of glycyrrhetinic acid lies in the ketone group at the C11 atom. Oleanolic, maslinic, arjunolic and echinocystic acids differ from each other in the number and location of additional hydroxyl groups. While oleanolic acid has only one hydroxyl group at C3, maslinic and echinocystic acids possess a second hydroxyl group at the C2 and C16 positions, respectively. Furthermore, arjunolic acid features a third hydroxyl group at C23 (Figure 2). Structural modifications of pentacylic triterpenoid acids with the oleanane skeletal type have focused mainly on the functional carboxylic and/or hydroxyl groups, due to the relative chemical inertness of the triterpenoid core. For instance, the hydroxyl group is often utilized for esterification reactions with carboxylic acids, acid chlorides, or anhydrides to form various esters or glycosylation reactions to introduce sugar moieties, which often improve the water solubility of pentacyclic triterpenoid acids [14]. Another popular modification for the hydroxyl group is its transformation into a ketone using Jones reagent or other strong oxidants [14,15]. The carboxylic acid group is also a well-established site for introducing novel structural motifs to pentacyclic triterpenoid acids. Esterification and amidation reactions are the most common reactions utilized to transform this active group and add new structural motifs [14,16]. Carboxylic group was also reported to be utilized for the copper-catalyzed azide-alkyne cycloaddition (CuAAC), a famous click chemistry reaction allowing the formation of triazole analogs [17,18]. Another location, which can be utilized to modify the pentacyclic triterpenoid acids with the oleanane skeletal type, is the endocyclic double bond of the C-ring of the core structure. For instance, acetylation of the C-3 hydroxy group, followed by mCPBA-mediated epoxidation of the C12–C13 double bond and intramolecular opening of the resulting epoxide by the C-28 carboxyl group, affords 3β-acetoxy-12α-hydroxyoleanan-28,13β-olide. Subsequent oxidation of the C12 hydroxy group followed by oxime formation provides the corresponding 12-oxime. Treatment of this oxime under Beckmann rearrangement conditions affords a nitrile derivative retaining the 28,13β-lactone moiety and a C-ring-expanded lactam derivative [19]. Alternatively, fluorolactonization using Selectfluor introduces a fluorine atom at C-12 alongside lactone ring formation between C-28 and C-13 atoms [20]. A great number of derivatives of natural pentacyclic triterpenoid acids with the oleanane skeletal type capable of targeting breast cancer has been tested over the years. The most promising ones are summarized in Table 1.

### 2.1. Oleanolic Acid and Its Derivatives

**Figure 3:**
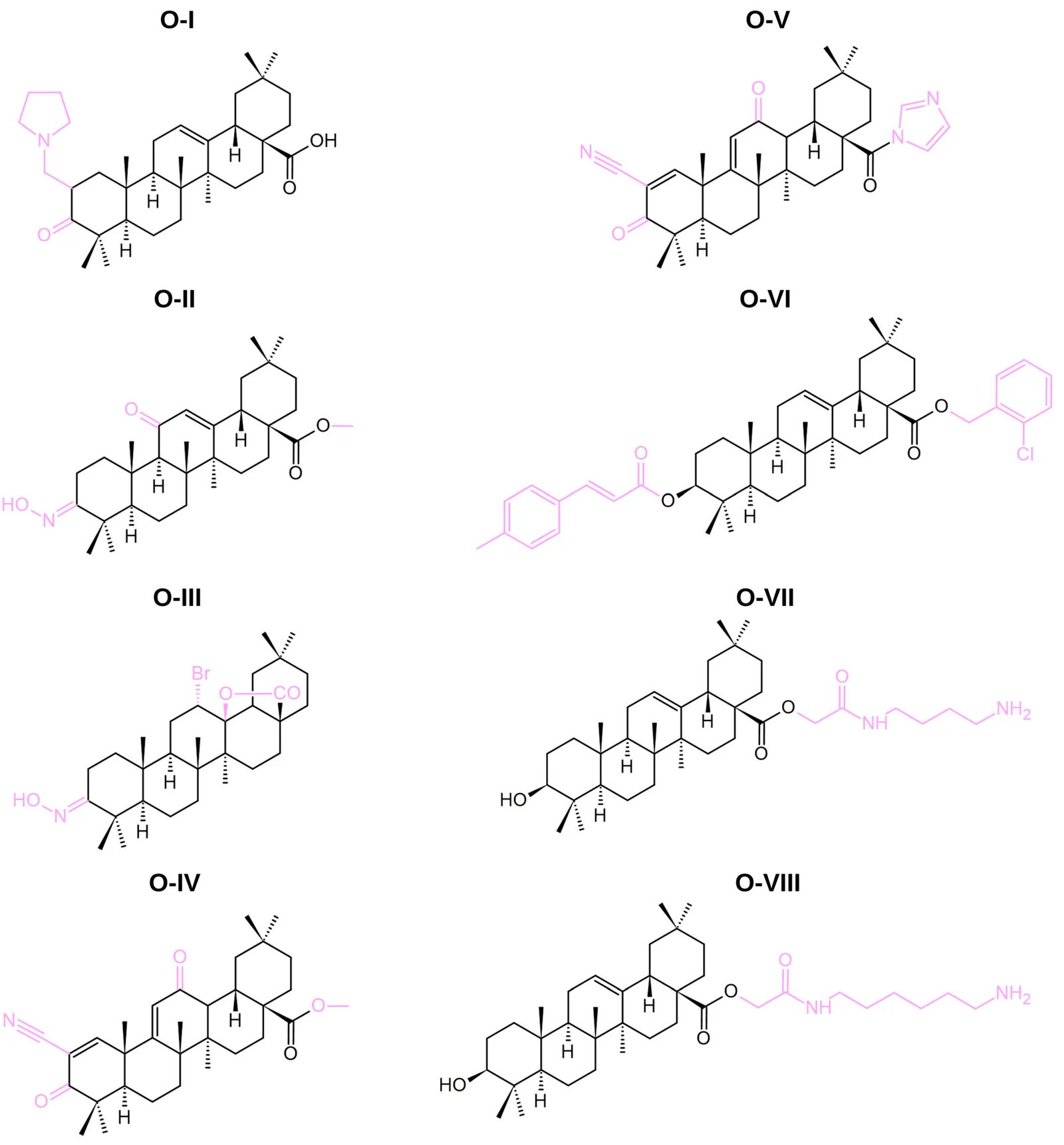
The structures of the derivatives of oleanolic acid. The structural differences between the compounds are highlighted in pink.

Oleanolic acid (OA) is a natural compound present in the leaves, roots, and stems of more than 1600 plants [39]. It has a wide range of biological activities, including anti-inflammatory, antioxidant, antimicrobial, wound healing, and especially anticancer. Its ability to inhibit oncogenesis is achieved via various molecular targets and mechanisms. Findings present that one of the key paths is apoptosis induction, a natural process of the body that targets cancer cells without causing significant inflammatory response, avoiding the inflammation associated with necrosis. Apoptosis is initiated via either an intrinsic or extrinsic pathway. The intrinsic, also known as the mitochondria-mediated, pathway is often associated with the disruption of mitochondrial membrane potential (MMP). In the case of OA, the intrinsic pathway of apoptosis is initiated by the increase in reactive oxygen species (ROS), decrease in anti-apoptotic B-cell lymphoma 2 (Bcl-2) protein and simultaneous increase in proapoptotic Bcl-2 Associated X-protein (Bax). This leads to the activation of caspase cascade via initiator caspase 9 and executioner caspase-3, which impair cell structures and cleave poly ADP-ribose polymerase (PARP), inhibiting the DNA reparation process. The extrinsic apoptotic pathway can also be induced by OA via an increase in caspase 8 as a result of initiation of death receptor signaling cascades. Moreover, Oleanolic acid also suppresses the Protein Kinase B (AKT) signaling pathway, further inhibiting cell survival and decreasing the breast cancer cells’ ability to resist apoptosis [40,41]. Oleanolic acid can induce autophagy through Akt/mTOR/S6K signaling pathway inhibition [42]. OA can cause an increase in autophagy-related LC3-II, Beclin-1, and p62/SQSTM1 proteins. Furthermore, visual verification of LC3 puncta formation confirms the production of autophagosomes as a result of oleanolic acid treatment. Moreover, the blockage of autophagy-related genes (*ATG5*, *ATG7*) and the use of the autophagy inhibitor 3-methyladenine decreases the cytotoxicity of oleanolic acid, showcasing a direct connection between autophagy induction and anticancer effects of oleanolic acid [43]. A number of studies demonstrate that oleanolic acid can also modify the cancer cells metabolism by regulating lipid and protein synthesis [44,45]. Such effects can be obtained by activating the AMP-activated kinase (AMPK) and therefore inducing aerobic glycolysis. AMPK activation coincides with the suppression of lipogenesis, protein synthesis, lactate production, and lowered glucose uptake. Another mechanism by which oleanolic acid affects cancer metabolism is inhibition of the mTOR/c-Myc/PKM2 signaling axis, switching pyruvate kinase muscle isozyme 2 (PKM2) to PKM1. These changes increasingly induce oxygen uptake, which limits energetic capacity and biosynthesis opportunities in the anaerobic environment, reducing the capabilities of cancer cells to grow and multiply [45]. Oleanolic acid was among the most popular pentacyclic triterpenoid acids for synthetic modifications (Figure 3).

One of these derivatives, compound **O-I,** published by Chu et al. [21], has a pyrrolidine fragment connected to the C2 atom of the oleanane core. The primary mechanism of anticancer activity of **O-I** is via ROS generation, which causes the initiation of the intrinsic pathway of apoptosis, as can be noted by elevated levels of caspases 9 and 3. Moreover, the utilization of antioxidant compound N-acetylcysteine significantly attenuated the proapoptotic effects of **O-I** in MCF-7 cells, showcasing a direct connection between elevated ROS levels and proapoptotic activity of the novel compound. This novel compound exhibited potent effects towards cell cycle arrest, as was confirmed by increased G1 phase of the cell cycle post-treatment, cyclins D1 and CDK4/6 downregulation, and increased levels of p21 and p27 proteins. Finally, **O-I** inhibited colony formation and cell migration [21]. **O-II** and **O-III** are semisynthetic derivatives of oleanolic acid, modified at the C3, C11 and C28 positions that Lisiak N. et al. [22] evaluated based on in vitro tests on human epidermal growth factor type 2 (HER-2)-positive breast cancer cells SK-BR-3. Based on molecular docking, the authors predicted that these compounds could bind with the HER-2 receptor and potentially modulate its signaling. MTT assay was utilized to confirm the cytotoxic abilities of these compounds, with IC_50_ values equal to 3.89 ± 0.28 µM after treatment with **O-II** and 3.87 ± 0.35 µM with **O-III** after 24 h. Obtained derivatives exhibited significantly more potency than the parent compound, with IC_50_ equal to 22.5 ± 1.27 µM. Furthermore, several tests exploring molecular mechanisms of action were conducted. Obtained data revealed that compounds arrested cells in G0/G1 phase with decreased S phase in comparison to the control. It should be noted that **O-III** induced an increase in the apoptotic cell population, while **O-II** did not indicate such capability. Furthermore, **O-III** also increased proapoptotic proteins levels (Bax, cleaved PARP), simultaneously decreasing the HER2 targeted protein. In contrast, **O-II** did not display such effects. Nonetheless, both compounds induced autophagy, as was confirmed by monodansylcadaverine (MDC) staining, which demonstrated that more acidic autophagic vesicles formed. Moreover, the concentrations of the autophagosome formation marker LC3-II/LC3-I ratio were increased, and beclin-1 levels were increased, while p62 and mTOR were decreased, supporting the fact that the autophagy process was activated. Finally, both structures reduced cell migration by 95 and 80% post-treatment with **O-III** and **O-II**, respectively. The authors reported that the effects of tested structures on cell migration were performed by modulating the integrin β1/FAK (Focal Adhesion Kinase)/paxillin pathway. Further research into the anticancer effects of those two structures was performed by Lisiak N. et al. [23]. In their new work, the authors described **O-II**’s and **O-III**’s influence on estrogen receptor (ER) and Epidermal Growth Factor Receptor (EGFR)-mediated cancer cells. The authors assessed how a particular receptor expression can affect the anticancer activity of their OA derivatives. Against MCF-7 cells with ER^+^ expression, IC_50_ value after treatment with **O-II** was 3.22 ± 0.42 µM and with ER^−^ expression was 1.63 ± 0.1 µM. In the case of **O-III** on ER^+^ cells, IC_50_ value was equal to 6.58 ± 0.74 µM and 3.53 ± 0.4 µM against ER^−^ cells. The cytotoxicity measurement against the MDA-MB-231 cell line with EGFR^+^ and EGFR^−^ expressions was performed as well. **O-II** revealed IC_50_ equal to 21 µM against cells with EGFR^+^ expression and 1.72 ± 0.081 µM EGFR^−^ expression. Interestingly, **O-III** only showed cytotoxic activity against EGFR^−^ cells (IC_50_ = 3.44 ± 0.19 µM), not affecting EGFR^+^ cells. Cell cycle analysis showed that both compounds arrested cells in the G0/G1 phase, simultaneously decreasing cell population in S and G2/M phases, respectively, in MCF-7 ER^−^ cells. However, only **O-II** managed to increase apoptotic cell population in MDA-MB-231 EGFR^+^ cells. Based on their previous work, the authors studied the autophagy-inducing capabilities of the two structures. **O-II** managed to increase autophagy only in cells with ER^+^ and EGFR^+^ expressions, while **O-III** also demonstrated such capability in both ER^−^ expression and EGFR^−^ breast cancer cell lines. The authors also assessed levels of molecules involved in cell adhesion. Integrin β1, FAK, Tyr397FAK, total FAK and paxillin levels were analyzed with Western blot and supported the fact that **O-II** and **O-III** reduced potential migration and invasion of cells [23]. The findings presented in these publications show that **O-II** and **O-III** exhibit anticancer potential on several breast cancer cell lines and indicate clear differences in their anticancer effects and cell death modulation depending on the influence of ER and EGFR, which, however, need to be studied utilizing in vivo research methods for confirmation of such effects in complex and full biological models (Figure 3).

Through a series of structural modifications of OA, including the formation of a methyl ester at C-28, the derivative **O-IV** was obtained [27]. This molecule is capable of modulating tumor progression mostly by downregulated infiltration of tumor-associated macrophages (TAMs) into tumors. Michael S. Ball performed in vivo experiments on PyMT^+/−^ female mice, which proved that after utilizing this, molecule markers associated with TAM were decreased. Low concentrations of CD206 and CD115 were equivalent to the inhibition of pro-tumor macrophage phenotypes. Moreover, **O-IV** affected signaling between immune cells and TME, reducing CCL2 and CXCL16 proteins level. When these chemokines remain in low concentrations, then macrophages’ abilities to recruit and reshape trafficking cells among TME are significantly reduced. In addition, researchers performed ELISA (enzyme-linked immunosorbent assay) to evaluate **O-IV** influence on various tumorigenic cytokines. The following data were obtained: IL-10 (interleukin-10) and VEGF (vascular endothelial growth factor) levels were decreased, suggesting that immunosuppressive and angiogenic potential of tumors were reduced. Moreover, TNF-α (tumor necrosis factor alpha) levels were increased, killing the tumor cells. This paper revealed anticancer potential of **O-IV** utilizing in vivo research [25]. Another mechanism of activity researched for the **O-IV** compound was inhibition of the Wnt/β-catenin pathway. In physiological conditions, the Wnt/β-catenin signaling pathway allows cell differentiation and proliferation to be maintained. However, in the case of cancer, this path can be utilized to promote tumor progression. When the Wnt ligand is present, it can bind with the LRP6 receptor, then activate the Dishevelled protein (DVL) and cause β-catenin accumulation in the cytoplasm, supporting cell adhesion. The authors performed tests on a murine xenograft model, administrating **O-IV** to mice bearing mammary tumor virus (MMTV). Results showed that this compound reduced expression of LRP6, DVL2 and active β-catenin, confirming its effects against this mechanism of cancer survival [24]. Refaat A. et al. [26] also analyzed the anticancer effects of **O-IV**. However, in their work, the authors focused on its effects on ROS-generating capabilities of this compound. **O-IV** can activate the Nrf-2 (nuclear factor erythroid 2-related factor 2) signaling pathway responsible for transcription of the antioxidant gene, which causes the decrease in intracellular glutathione levels (GSH) and reduced expression of the antioxidant *SOD2* gene, leading to the build-up of mitochondrial ROS concentration. Moreover, pro-survival signaling was also inhibited. Western blot analysis showed that this derivative decreased AKT phosphorylation, NF-κB signaling, and Mitogen-Activated Protein Kinase (MAPK) pathways. Researchers confirmed reduced migration of MCF-7 cells with scratch assay. Data obtained in this article showed that metabolic reprogramming induced by **O-IV** is an important mechanism of the anticancer activity of this structure (Figure 3) [26]. In the manuscript by J. Young, the antitumor properties of compound **O-V** were evaluated in triple-negative breast cancer (TNBC) models. To synthesize this analog, an imidazolide group was introduced at the C28 position of CDDO. Experiments were conducted on SUM159 and MDA-MB-231 cell lines; the results obtained by the authors associated anticancer potential with the regulation of stem cell signaling pathways. In this article, TNBC cell growth was inhibited after administration of **O-V**. In the SUM159 cell line, the amount of CD24−/EpCAM+ was reduced, lowering the efficiency of tumorspheres forming and decreasing the tumorsphere size. Moreover, the authors noted that **O-V** inhibited pathways important in maintaining stem cells such as Notch, transforming growth factor beta (TGF-β)/Smad, Hedgehog and Wnt pathways. Decreased activity of proteins involved in these pathways supports disrupted communication between cancer stem cells and may lead to cell cycle arrest and apoptosis. In this study, the cell cycle was arrested in the G2/M-phase and the number of apoptotic cells was significantly increased after treating the cells with this analog. However, the experiments described in this article were performed in an in vitro model, and it is crucial to test these effects in in vivo models [28]. Wang et al. [29] designed and synthesized a series of oleanolic acid–cinnamic acid ester derivatives and glycyrrhetinic acid–cinnamic acid ester derivatives. The compounds were modified at both the C-3 hydroxyl and carboxyl groups through the introduction of cinnamoyl and benzyl ester moieties, respectively. Structural diversity was achieved by incorporating fluoro-, chloro-, bromo-, and methyl-substituents into the aromatic rings, enabling evaluation of structure–activity relationships and cytotoxic activity. Cytotoxic activity of novel compounds was evaluated on three cell lines: MCF-7, HeLa and L-O2. On the breast cancer line, the compound **O-VI** showed the strongest inhibitory activity with an IC_50_ value of 1.79 μM; however, the mechanism of action was not assessed [29]. A novel group of OA derivatives was developed by Tian T. et al. [30]. The authors synthesized a series of eighteen analogs of oleanolic and ursolic acid (UA) by esterification at the C-28 position with 2-hydroxyacetic acid, followed by amidation with several amines afterwards. New compounds were tested on three cancer cell lines: MCF-7, Hela and A549. Compounds **O-VII** and **O-VIII** with introduced butyl- and hexylamine, respectively, demonstrated the most promising potential against the breast cancer cell line with the following IC_50_ values: 7.48 ± 0.33 µM and 7.61 ± 0.33 µM. Interestingly, oleanolic acid derivatives slightly suppressed ursolic acid derivatives with identical structural modifications. Furthermore, the elongation of the carbon chain of the amine substituent at C28 provided a significant increase in anticancer activity. Molecular mechanisms of action were not evaluated in this paper [30].

### 2.2. Maslinic Acid and Its Derivatives

**Figure 4:**
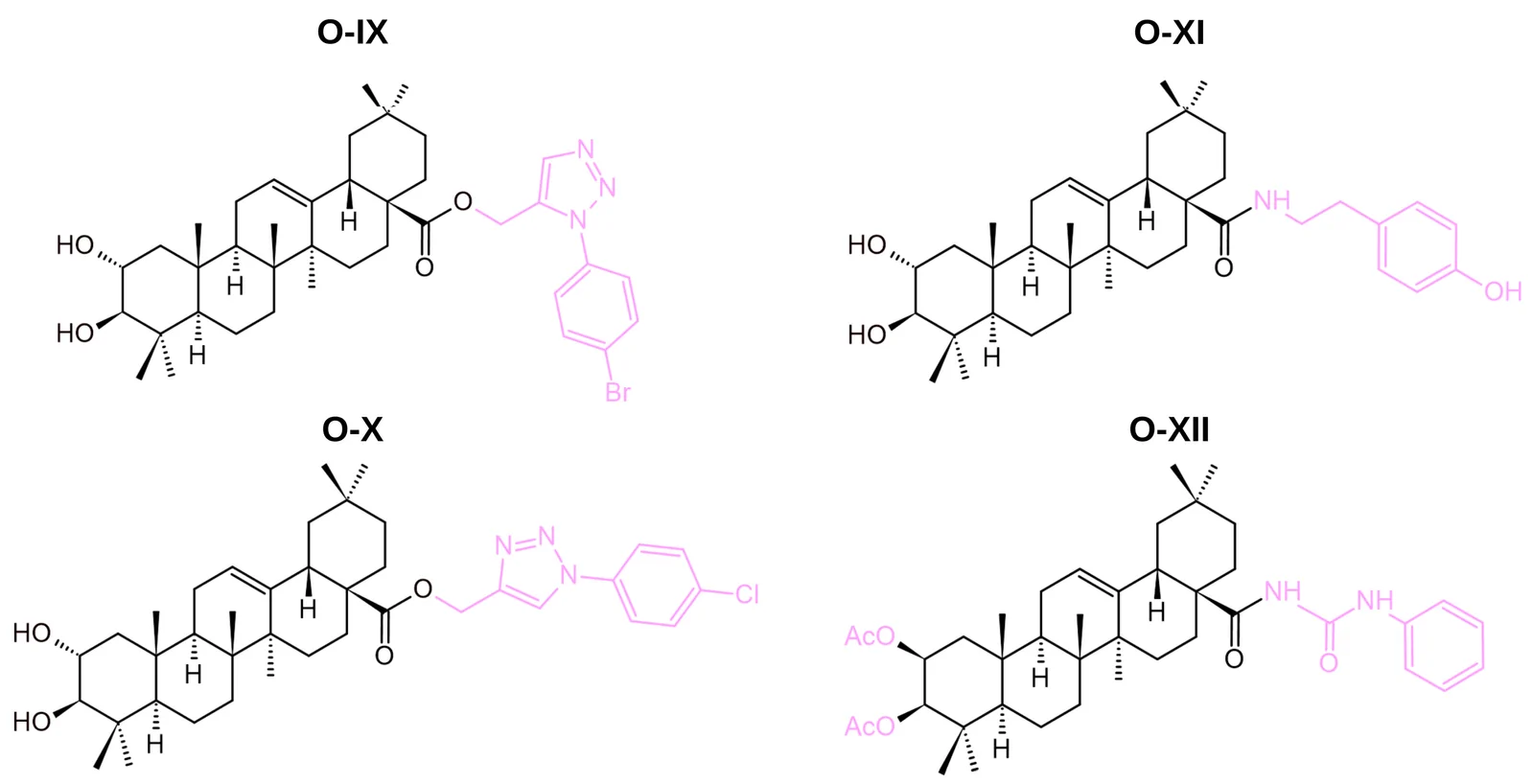
The structures of maslinic acid derivatives. Structural differences between the compounds are highlighted in pink.

Maslinic acid (MA) belongs to the pentacyclic triterpenoids with the oleanane skeleton group. It is naturally present in such plants as *Olea europaes* L. and *Crategus pinnatifida* Bunge. MA exhibits antidiabetic, analgesic, antibacterial, antiviral, cardioprotective, and antiparasitic activity [46,47,48,49,50]. The anticancer effects of maslinic acid were also investigated by Jain et al. [46]. The authors showed that this acid inhibited breast cancer cell proliferation, with the MTT test obtaining the following IC_50_ values: 38 and 34 µM on the MDA-MB-231 cell line; 49 and 57 µM on the MDA-MB-468 cell line, and 55 and 20 µM on the MCF-7 cell line, with no noticeable toxic effects on non-cancerous fibroblasts [46]. MA mechanisms of action can vary depending on the presence of estrogen receptors. For instance, in TNBC cells with estrogen receptors, MA caused cell cycle arrest at the G0/G1 phase, with decreased expressions of cyclin-dependent kinase 2 (CDK-2), CDK4, and a downregulated MAPK/ERK pathway. In contrast, the ER-positive MCF-7 cells were arrested at S and G2/M phases of the cell cycle, while the expression of CDK-2 was increased and MAPK/ERK elevated. Nonetheless, all treated cell lines displayed the reduction of MMP and caused caspase-independent apoptosis. Furthermore, maslinic acid reduced reactive oxygen species generation, a notable difference from many other pentacyclic triterpenoid acids [46]. Finally, another article by K. Wang provided confirmation of the supporting role of maslinic acid in cancer treatment. Obtained data displayed that it can enhance docetaxel activity by increasing cell sensitivity and reducing drug resistance [51]. A number of authors researched the possibility of structural modification of maslinic acid (Figure 4) [31,32,33].

For instance, Chouaïb K. described 40 derivatives of maslinic acid bearing 1,5- and 1,4-disubstituted triazoles. His team synthesized a group of new analogs under microwave conditions. First, propargylation at C-2/C-3 or C-28 of MA was performed, followed by RuAAC or CuAAC click chemistry reactions, forming novel triazole derivatives with different phenyl substituents. Researchers evaluated anti-inflammatory and antiproliferative activities of novel compounds on two cell lines: EMT-6 and SW480. Compound **O-IX**, with the 4-bromophenyl group substituted in the N-1 position and maslinic acid ester attached to the triazole ting in the C-5 position, decreased cell viability to 13%. Compound **O-X**, with the 4-chlorophenyl group substituted in the N-1 position and a maslinic acid-derived fragment attached to the triazole ring in the C-4 position, reduced cell viability to 6% at the concentration of 30 μM. From all tested compounds, these derivatives presented the strongest antiproliferative properties. However, a detailed analysis of the mechanism of anticancer activity of those structures is required in future studies [31]. Fuentes-Rios et al. [32] synthesized and evaluated another group of maslinic acid analogs. In this paper, MA was conjugated with glycerin, tyramine oligo and amino acids to overcome existing limitations in solubility and anticancer activity. Six new derivatives were tested against a wide range of various cancer cell lines, among which two were breast cancer: MCF-7 and MDA-MB-231. Out of all tested compounds, the structure **O-XI**, a tyramine-maslinic acid conjugate with the newly introduced fragment present at the C28 atom of maslinic acid, showed the strongest cytotoxic activity with four times more potent effect than the original MA in MCF-7 cells. The IC_50_ value of **O-XI** was 15.71 ± 1.86 μM, where MA IC_50_ was 64.8 ± 4.14 μM. **O-XI** was 2-fold more active against MDA-MB-231 cells, where **O-XI** IC_50_ was 17.98 ± 1.00 μM and MA IC_50_ was 32.65 ± 3.86 μM. Furthermore, the authors measured the solubilities of the obtained derivatives since it is one of the main problems of the original pentacyclic triterpenoid acid. The most active structure, **O-XI**, exhibited relatively mild solubility in water with 0.2 mg/mL. However, in other polar solvents the obtained data is much better. For example, **O-XI** solubility in methanol and propyleneglycol was equal to 9 mg/mL. Overall, it is an improvement from the original MA in both its cytotoxicity and bioavailability. Nonetheless, the obtained structure requires more in-depth research regarding its mechanism of activity and safety profile [32]. In another paper, Sommerwerk S. et al. [33] evaluated urea derivatives of ursolic, oleanolic, and maslinic acids. The researchers protected the hydroxyl groups at the C2 and/or C3 atoms with acetyl groups to safely introduce the urea moieties at the C-28 atom. Interestingly, the most promising compound among those synthesized was **O-XII**, with 2β,3β-configurated centers and phenylurea moiety at C28. Compound **O-XII** exhibited 2.8 μM on MCF-7 and >120 μM NIH 3T3. **O-XII** was chosen for further investigation against the A2780 ovarian cell line, since the structure was more potent against it. While research revealed apoptosis induction against that cell line, the analysis of proapoptotic effects against breast cancer cell lines is necessary to confirm it [33].

### 2.3. Glycyrrhetinic Acid and Its Derivatives

*Glycyrrhiza* species (licorice) are commonly known in Europe and Asia. For thousands of years, they were extensively used as herbal medicine and sweets. *Glycyrrhiza glabra*, *Glycyrrhiza inflata* and *Glycyrrhiza uralensis* are examples of such plants. Pharmacological properties of licorice have been evaluated as antiviral, analgesic, anti-inflammatory, antioxidant, and antitumor [52,53,54,55].

Glycyrrhizic acid (GL) converts to 18β-glycyrrhetinic acid (18β-GA) as a result of human intestinal microflora, and its activity has been described as proapoptotic and antimetastatic. It can also activate autophagy, which can be both protective and cytotoxic on cancer cells. Yu-Chih Hsu et al. [55] described effects of 18β-GA in MCF-7 and T-47D cell lines, estimating 125.8 µM and 135.6 µM IC_50_ values after 48 h post-treatment. Further research revealed that at 100 µM it caused G0/G1 cell cycle arrest and at 150 µM it exhibited caspase-dependent apoptosis induction and PARP cleavage. Moreover, 18β-GA caused an increase in LC3-II and p62 accumulation which impaired autophagosome degradation. Another mechanism of anticancer activity was the c-Jun N-terminal kinase (JNK) signaling pathway. The researchers proved that treatment with this compound improved JNK phosphorylation, and inhibition of this process via the JNK inhibitor (SP600125) was linked with a reduction of LC3-II accumulation, reduction in apoptotic population, and restored colony formation. This suggests that JNK activation is related to autophagy modulation and apoptosis induction by 18β-GA. Finally, the authors applied in vivo tests on an MCF-7 xenograft mouse model. A total of 25 mg/kg of 18β-GA were administered, and tumor growth was significantly reduced, with no toxic effects on liver and body weight loss noted [55]. 3-Acetyl-18β-glycyrrhetinicacid-30-methyl ester (**O-XIII**) is a semisynthetic analog of glycyrrhetinic acid evaluated by Mohamed G. Ibrahim and his team. In the study, a derivative was tested on MCF-7 breast cancer cells and cytotoxic activity was noted, with lower toxicity on WI-38 normal lung fibroblasts. On MCF-7 cells, the IC_50_ value was 4.5 ± 0.1, and on WI-38, the IC_50_ value was >50. Cytotoxic effects may be the result of the presence of the acetyl group at C-3 and the methyl ester group at C-30 as confirmed by SAR assessment. The article shows that **O-XIII** elevated the number of apoptotic cells, increased the p53 proapoptotic protein, and induced cell cycle arrest. Moreover, this compound inhibited clonogenic survival and cell migration, which prevents metastasis. However, the mechanism of these effects was not evaluated [34]. In his paperGaber O. Moustafa described new 18β-GA peptide derivatives with introduced peptide fragments at the C28 atom of original pentacyclic triterpenoid acid. New structures were tested for their cytotoxic activity against MCF-7, HCT-116, HepG-2 and BJ cell lines. Among them, **O-XIV,** bearing a tetraglycine fragment at C-30 with a free terminal carboxylic acid, exhibited the most promise due to its selective activity against MCF-7 breast cancer cells (IC_50_ = 5.0 ± 0.3 µg/mL), with only minor cytotoxicity toward normal BJ-1 cells (20%), and was therefore chosen for further in-depth analysis. **O-XIV** increased Bax/Bcl-2 ratio and p53, which suggests a mitochondrial pathway of apoptosis, with the increase in the concentrations of caspases 3 and 7 leading to cell death. Interestingly, the compound exhibited the ability to inhibit β-tubulin polymerization, a well-known mechanism of activity of numerous FDA-approved drugs such as colchicine or vinblastine yet never mentioned in the context of pentacyclic triterpenoid acid [35]. A few years later, Gaber O. Moustafa, with his team, obtained another group of peptide glycyrrhetinic acid analogs. In their novel work, the newly synthesized derivatives demonstrated cytotoxic activity on the MCF-7 cell line. Notably, their effects against normal human fibroblasts, the BJ-1 cell line, were less toxic. Compound **O-XV** exhibited the most promising anticancer activity and was analyzed in greater detail. Cytotoxic assay presented that on MCF-7 cells, the IC_50_ value for this compound was 6.20 ± 0.25 μM. An increase in proapoptotic proteins, such as Bax, caspase-3, caspase-7, and p53, after treatment with compound **O-XV** was noted. Simultaneously, anti-apoptotic Bcl-2 protein level was decreased. This derivative increased the ratio of DNA fragmentation and was tested as a potential β-tubulin polymerization inhibitor; however, it did not suppress this process as effective as reference drug colchicine. Moreover, molecular docking revealed that compound **O-XV** has wider anticancer properties. It presented that it can potentially bind with CDK-2, regulating DNA synthesis and cell cycle progression. Another promising target for compound **O-XV** is VEGFR-2, a crucial receptor in angiogenesis. Platelet-derived growth factor receptor-α (PDGFR-α) also seems to be a potential target for compound **O-XV**, and its inhibition could reduce interactions between cancer cells. Results obtained from molecular docking only present the potential of this new analog and require further research to confirm these possibilities [36].

**Figure 5:**
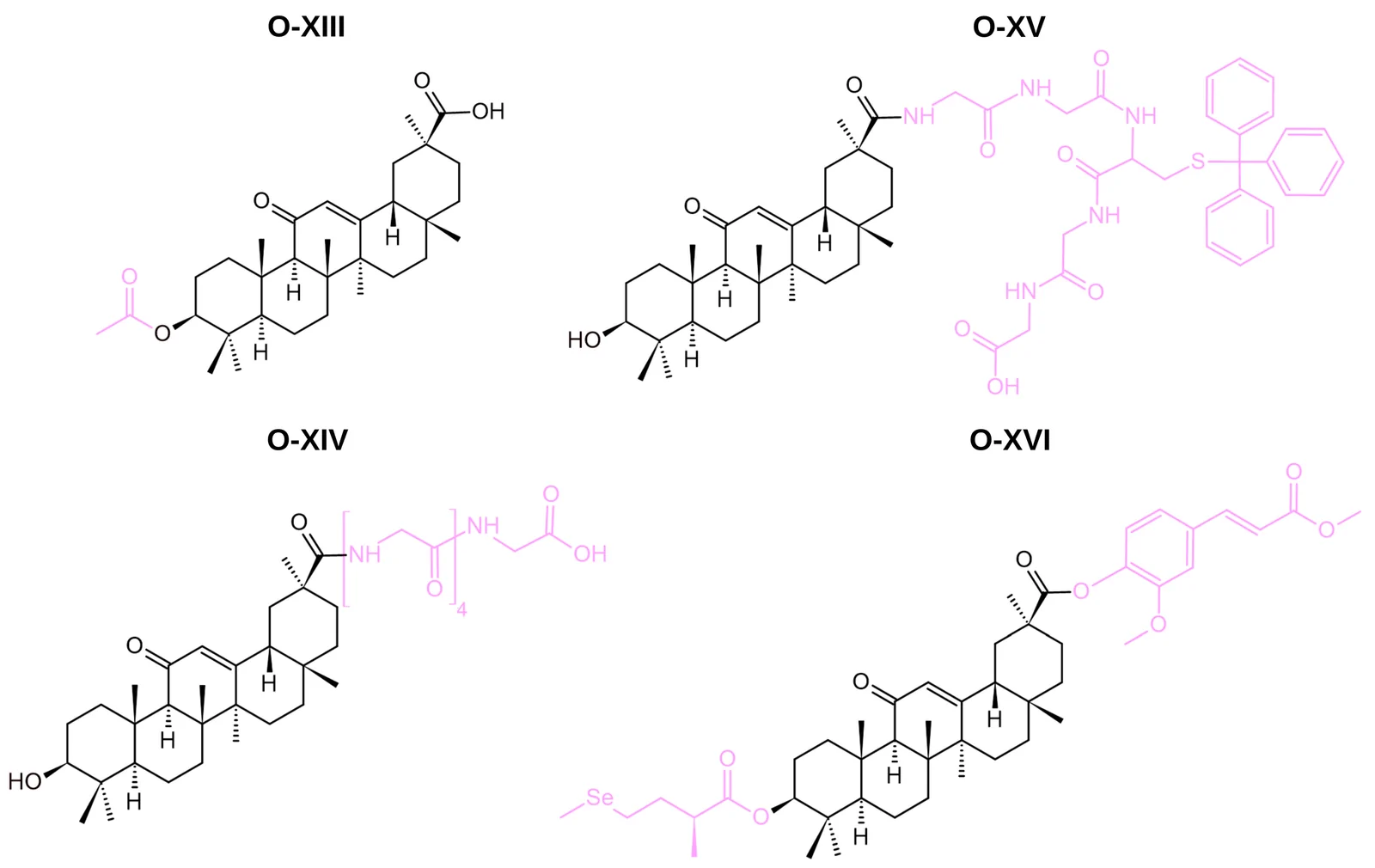
The structures of glycyrrhizic acid derivatives. Structural differences between the compounds are highlighted in pink.

Another group of derivatives was discovered by Li Y. et al. [37]. The researchers synthesized 40 analogs; they attached lipophilic fragments to the carboxylic group or different amino acids on the 3-hydroxy group to improve the cytotoxicity of the parent compound. The researchers noted that ferulic acid methyl ester analogs had stronger anticancer activity. Moreover, the addition of L-selenomethionine at the C-3 position significantly lowers IC_50_ values in comparison to L-methionine. An example of such activity is compound **O-XVI**, which presents the strongest anticancer effect against MCF-7 and MDA-MB-231 cell lines, estimating the following IC_50_ values: 1.88 ± 0.20 μM and 1.37 ± 0.18 μM. This paper focused on how chemical modifications of the parent compound can enhance the biological activity of 18β-GA derivatives (Figure 5), leaving a gap to fill with research evaluating molecular mechanisms of anticancer activity [37].

### 2.4. Arjunolic Acid

Arjunolic acid (AA) is another pentacyclic triterpenoid acid featuring the oleanane skeletal type. In previous years, arjunolic acid has been researched for its anticancer effects against breast cancer [56,57]. In an article by Manna et al. [56], the authors explored a self-assembling arjunolic acid for breast cancer treatment. To prepare the self-assembling particles, authors mixed the extracted AA with the solution of dimethyl sulfoxide (DMSO) and water in a 16:4 ratio, which allows unique molecular interactions between AA atoms, creating nano-sized and hollow vesicular structures, resembling liposomes. MTT assay showcased that MCF-7 cell viability was reduced by 66.98% at a concentration equal to 50 μg/mL. The self-assembled AA showed a favorable safety profile even at 100 μg/mL dosage, with 88.76% of viable lymphocytes post-treatment. The researched mechanism of action revealed that self-assembled AA provides its anticancer effects by elevating ROS levels. Furthermore, the glutathione levels decreased by 8-fold with a simultaneous 5-fold increase in oxidized glutathione. The tested structure also provided an increase in proapoptotic proteins such as interleukin-12 (IL-12) and TNF-α, while also decreasing antiapoptotic proteins such as IL-10 and TGF-β. To confirm the relation of the ROS-generating mechanism of activity, the authors pre-treated MCF-7 cells with N-acetyl-L-cysteine, which reduces the capability of ROS to affect cells. This reduced cell death by up to 47.45%. This suggests a multi-targeting mechanism of activity, and other possible molecular targets should be researched in the future [56]. Another article featuring anticancer activities of arjunolic acid against breast cancer was published by Akamse et al. [57]. The authors collected the stem bark from *Terminalia ivorensis* and isolated five compounds, including lupeol, betulinic acid, arjunolic acid, 3,3′-Di-O-methylellagic acid-4′-O-β-D-glucopyranoside, and 3′,3′,4′-Tri-O-methylellagic acid-4-O-β-D-glucopyranoside, and tested them against three different breast cancer cells (MCF-7, MDA-MB-231, 4T1). Arjunolic acid revealed the lowest CC_50_ values equal to 22.5, 25.4 and 18.3 µg/mL for MCF-7, MDA-MB-231 and 4T1 cell lines, respectively. To further confirm the potent effects of AA against breast cancer, the authors performed extensive in vivo assessment in comparison to untreated mice and mice treated with tamoxifen. Arjunolic acid at a concentration equal to 1 mg/kg displayed an 89% reduction in tumor burden, tumor incidence equal to 12.5%, and ~95% tumor mass reduction in comparison to the untreated control. Such results outperformed the clinically applied drug tamoxifen. Furthermore, AA exhibited anti-inflammatory properties as well by decreasing numerous inflammation proteins (TNF-α, IFN-γ, IL-6, and VEGF). Finally, kidney and liver markers (ALT, AST, ALP, urea, bilirubin, and creatinine) were also decreased in comparison to untreated mice with tumors and mice treated with tamoxifen. The article by Akamse et al. [57] provided ample evidence towards the promising anticancer activity of AA and its safety profile, utilizing both in vitro and in vivo methods.

### 2.5. Echinocystic Acid and Its Derivative

Echinocystic acid (EA) is naturally occurring pentacyclic triterpenoid acid, with the oleanane skeletal type, present in numerous plants such as *Albizia julibrissin*, *Gleditsia sinensis*, *Codonopsis lanceolata*, *Acacia ligulata*, and many others. In the last decade, only two articles have featured echinocystic acid and its derivatives targeting breast cancer [38,58]. In an article by Garai et al. [58], authors performed the assessment of antimicrobial and anticancer properties of the EA itself, extracted from *Luffa cylindrica*. The authors analyzed the cytotoxic properties of echinocystic acid against MCF-7 and MDA-MB-231 breast cancer cells and measured IC_50_ values equal to 41.72 and 48.17 µM, respectively, for those two cell lines. Furthermore, the authors analyzed those two concentrations against isolated normal human peripheral blood mononuclear cells and obtained promising results, with 96.29 and 84.34% of cell viability, respectively. Nonetheless, the article did not provide an in-depth analysis of the mechanism of anticancer activity. Taking into consideration high IC_50_ values and the lack of a detailed mechanism of anticancer activity, echinocystic acid requires more detailed analysis with a combined synthetic approach to introduce various structural modifications to improve the cytotoxicity of the compound [58]. A study by Tan et al. [38] introduced five new derivatives of echinocystic acid and tested them against standard and doxorubicin (DOX)-resistant MCF-7 cells. Among them, the **O-XVII** derivative with the ketone group instead of the hydroxyl group at C3, and the methoxycarbonyl group instead of the carboxyl group at C28, demonstrated the most potent cytotoxicity. The measured IC_50_ values were equal to 20.7 ± 2.7 and 31.1 ± 4.0 µM for MCF-7 and MCF-7/DOX, respectively. Perhaps the introduced structural modifications further contributed to the lipophilicity of the original EA, which increased the permeability of the derivative. Nonetheless, the authors did not test their derivative against normal cells, and the article did not provide in-depth analysis of the mechanism of the anticancer activity of EA or **O-XVII** [38].

## 3. Ursane Skeletal Type

**Figure 6:**
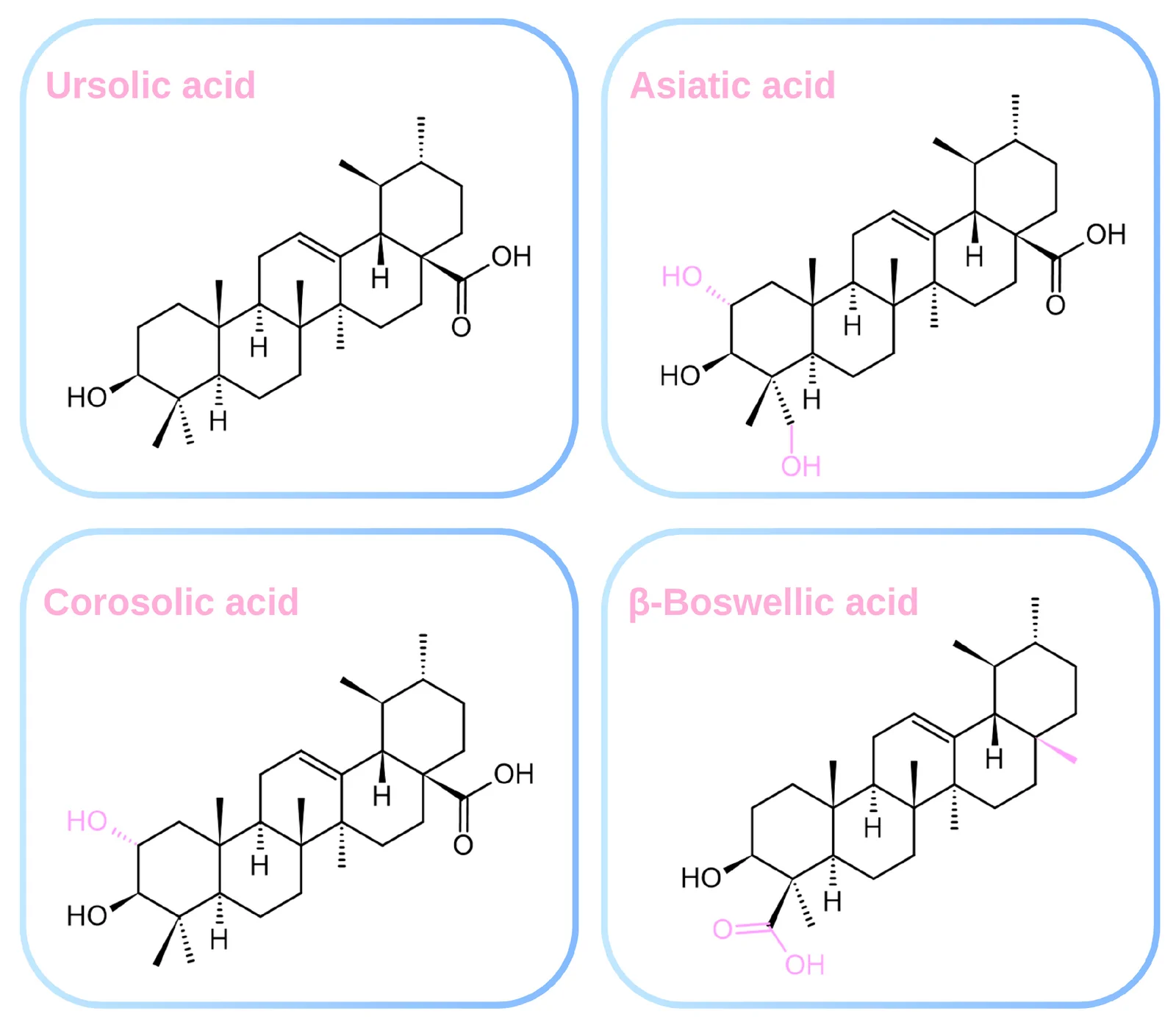
The structures of natural pentacyclic triterpenoid acids with the ursane skeletal type. The structural differences between the compounds are highlighted in pink.

The ursane skeletal type is similar to oleanane, except for the location of methyl groups at its E-ring. In contrast to the oleananes, ursanes have one methyl group connected to both the C19 and C20 atoms instead of two methyl groups at the C20 atom. In the last decade, four acids with the ursane skeletal type and their derivatives reported anticancer activity against breast cancer. Those are ursolic (UA), β-boswellic (BA), asiatic (ASA), and corosolic acids (CA). All of those compounds possess at least one hydroxyl group at the C3 position of the A-ring and a carboxylic group connected to either the C17 atom (ursolic, asiatic, corosolic acids) or C4 atom (β-boswellic acid). Corosolic and asiatic acids also have additional hydroxyl groups. In this regard, corosolic acid is similar to maslinic acid with a second hydroxyl group at the C2 atom of the ursane core, while asiatic acid is similar to arjunolic acid with a third hydroxyl group at the C23 atom (Figure 6). Considering the minor structural differences between oleananes and ursanes, the chemical modification of those acids does not significantly deviate from oleananes, focusing mainly on adding new structural fragments via reactions with hydroxyl and carboxylic groups [14,59]. Some of the more prominent derivatives of pentacyclic triterpenoid acids with anticancer activity against breast cancer are reported in Table 2.

### 3.1. Ursolic Acid and Its Derivatives

The main sources of ursolic acid are apples, cranberries, bilberries, peppermint, rosemary, oregano, and prunes. Due to its antioxidant and anti-inflammatory effects, research has been ongoing in recent years to investigate the effects of ursolic acid on cancer cells. Chakravarti et al., examining oleanolic and ursolic acids from *Wrightia tomentosa* extract on MCF-7 and MDA-MB-231 breast cancer cell lines, demonstrated that ursolic acid inhibits cancer cell proliferation and induces apoptosis by downregulating cyclin D1 and increasing Bax/bcl-2 ratio at concentrations of 5 and 7.5 µM across three time points of 24, 48 and 72 h [75]. Similar conclusions were established by Sathya et al., who conducted studies on the same cell lines and additionally on the T47D line, where inhibition of the EGFR pathway was observed, resulting in signal transducer and activator of transcription 3 (STAT3) phosphorylation and cell proliferation, as well as inhibition of the Phosphoinositide 3-kinase (PI3K)/AKT pathway and cell cycle arrest in the G0 phase [76]. Wang et al. [77] investigated the effect of UA from an extract obtained from *Oldenlandia diffusa* on inhibiting metastasis in the MDA-MB-231 cancer cell line. The study displayed that UA can significantly inhibit metastasis by activating the SP1/Cav-1 signaling pathway, which disrupts glycolytic metabolism. Additionally, the study demonstrated that UA inhibits the proliferation of both MCF-7 and MDA-MB-231 cancer cell lines by reducing the concentration of the antiapoptotic protein Bcl2 and increasing the proapoptotic protein Bax and cleaved PARP [77]. Zhao et al. [78] demonstrated on the MCF-7 cell line that UA also activates the autophagy-dependent gene EIF2AK3, which is a stress sensor of the endoplasmic reticulum. Studies have demonstrated that ER stress-dependent autophagy compromises the apoptotic effects of UA. This is associated with increased MCL-1 expression and induced cytoprotective autophagy through activation of MAPK1/3 [78].

**Figure 7:**
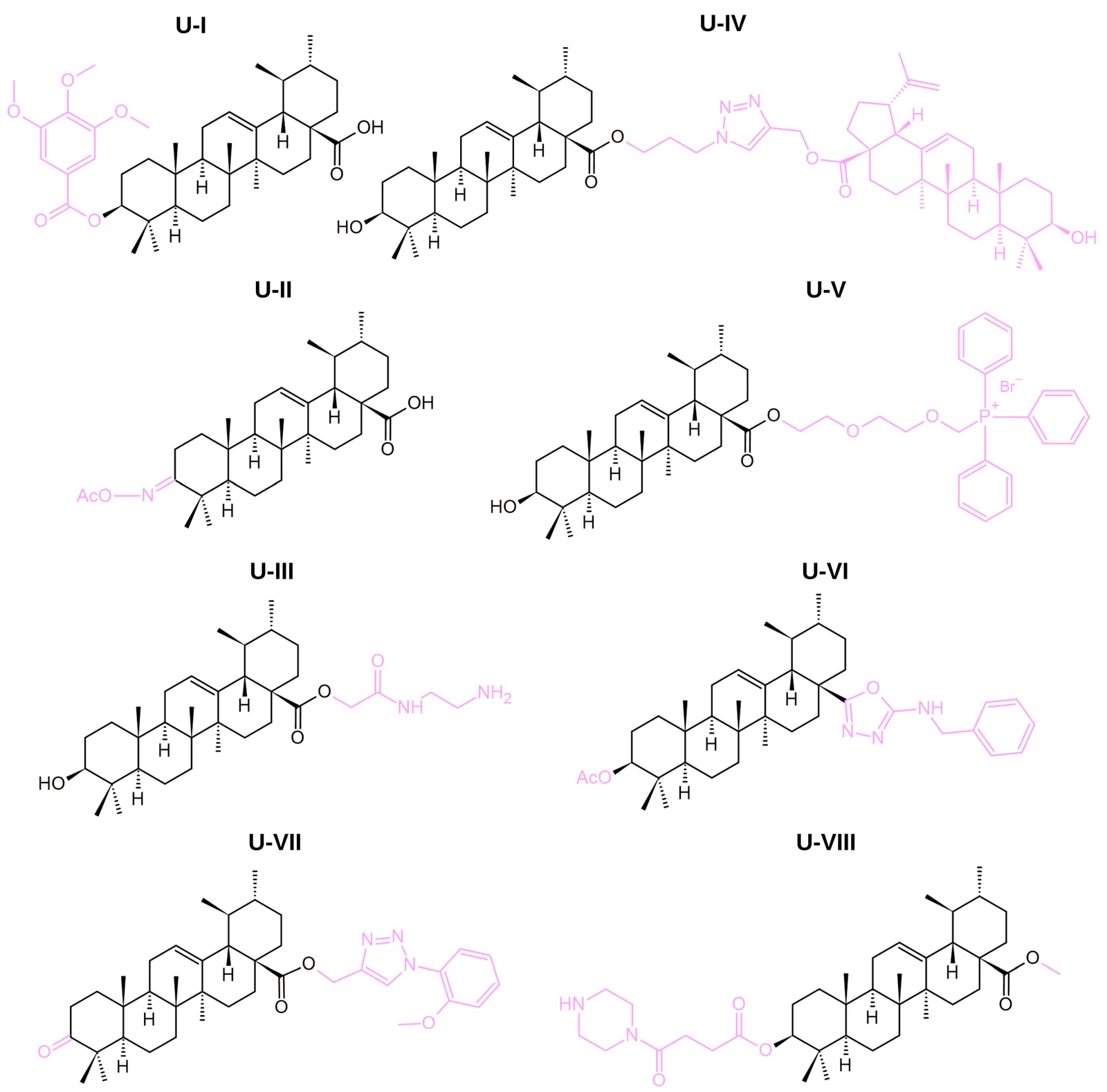
The structures of ursolic acid derivatives. Structural differences between the compounds are highlighted in pink.

Due to its poor bioavailability, which creates the consequent problem of appropriate administration of the substance, scientists are looking for synthetic modifications of UA to create derivatives that will increase the bioavailability of ursolic acid while maintaining its anticancer and anti-inflammatory properties (Figure 7). Thien et al. obtained UA derivatives by modifying the substituent at C3, replacing the hydroxyl functional group with a nitrosoacetyl group, where the IC_50_ concentration of the new derivative (**U-I**) was 3.43 µM, a 2-fold lower value in comparison to original ursolic acid, with IC_50_ equal to 8.0 µM for the MCF-7 cell line [60]. Xu et al. [61] designed a series of new compounds with a 3,4,5-methoxybenzoyl group at the C3 position of the UA structure to improve anticancer properties. This modification resulted in an increase in the cytotoxicity of the compound with IC_50_ decreasing from 51.87 to 22.27 µM on the MCF-7 cell line for the most potent derivative (**U-II**) [61]. Tian et al. obtained new derivatives, of which the most active was the one with the 1-(2-amino-ethylamino) substituent at the C28 atom of the pentacyclic triterpenoid core (**U-III**), where the IC_50_ value was equal to 8.45 µM in the MCF-7 cell line [30]. An interesting study was conducted by Pattnaik et al. [62], where the authors combined two different pentacyclic triterpenoid acids, namely ursolic and betulinic acid, utilizing a click chemistry reaction, a copper(I)-catalyzed azide-alkyne cycloaddition, creating an ursolic-betulinic hybrid connected via triazole (**U-IV**). The GI_50_ value was determined using the Sulforhodamine B (SRB) assay after 48 h and was equal to 14.4 µM for the MCF-7 line and 1.4 µM for the MDA-MB-231 line. Additionally, based on the study with Cyclin-B1, it was proven that the obtained compound changes the cellular phenotype and inhibits cells in mitosis [62]. Nedopekina et al. [63] conjugated UA with the lipophilic triphenylphosphonium cation (**U-V**) and tested its activity on the MCF-7 cell line. Compared to the cytotoxic effect of UA on breast cancer cells, the new analog exhibited an IC_50_ value of 1.59 µM in the MTT assay. Further research revealed that the novel derivative caused the intrinsic pathway of apoptosis [63]. Some of the studies displayed novel derivatives of ursolic acid with modifications at both C3 and C28 positions. Popov et al. [64] obtained derivatives with new substituents with an acetyl group at the C3 position and 2-amino-1,3,4-oxadiazole at the C28 position (**U-VI**). This modification afforded a derivative selective toward the MCF-7 line with potent cytotoxic activity and IC_50_ equal to 8.62 ± 2.33 µM [64]. Rashid et al. [65] synthesized a series of ursolic acid-1-phenyl-1H-[1,2,3]triazol-4-ylmethylester derivatives, in which the o-methoxy-substituted analog (**U-VII**) indicated the most potent cytotoxic activity against four different cancer cell lines, including breast cancer MCF-7 cells, with IC_50_ below 0.1 µM. Importantly, **U-VII** exhibited selectivity towards cancer cell lines, with little effects against FR-2 normal breast cells [65]. Zhao et al. developed structures, among which the derivative with a piperazine group at the C3 substituent and a carbomethoxy group at C28 (**U-VIII**) proved to be the most active, with an IC_50_ value at a concentration of 10.72 µM for the MCF-7 line after 48 h of incubation [66].

### 3.2. β-Boswellic Acid and Its Derivatives

**Figure 8:**
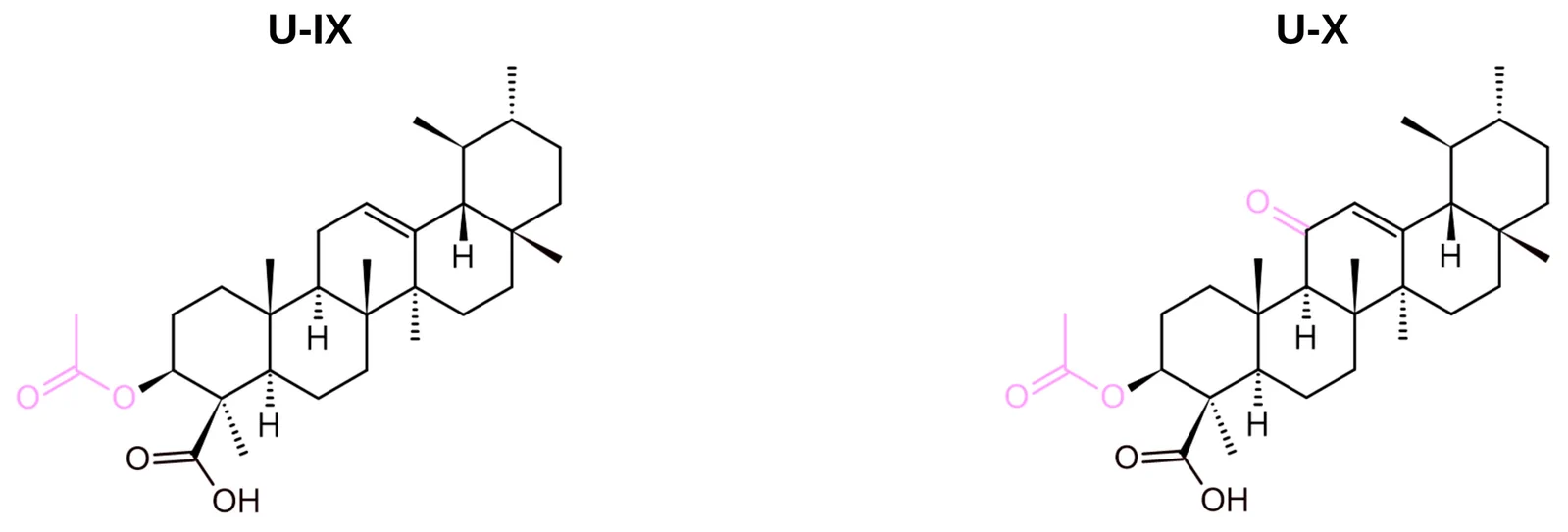
The structures of β-Boswellic acid derivatives. Structural differences between the compounds are highlighted in pink.

β-Boswellic acid belongs to the family of pentacyclic triterpene acids with a carboxyl group at C4 of the skeleton and is obtained from the *Boswellia* species. The most well-known derivatives of β-boswellic acid include 3-acetyl-β-BA (**U-X**) and 3-acetyl-11-keto-β-BA (**U-IX**) (Figure 8). These compounds are characterized by documented antidiabetic, antiviral, antibacterial, and anti-inflammatory effects [79]. Additionally, studies have been conducted to determine their anticancer properties. Ahmed et al. [67] revealed that **U-IX** tested on the MCF-7 cell line triggers the intrinsic mitochondrial pathway by producing reactive oxygen species, reducing mitochondrial membrane potential, and activating caspases 8 and 9, which ultimately leads to apoptosis. Additionally, they found that **U-IX** inhibits the cell cycle in the G1 phase. It should be noted that the cytotoxic effects were selective towards cancer cells, with no measured IC_50_ concentration against normal breast cells in MCF-10A [67]. In their studies, Zhang et al. [68] demonstrated that **U-IX** inhibits the concentrations of metalloproteinase-2 (MMP-2) and 9 in a dose-dependent manner (20, 30, 40 µM). This directly translates into a weakening of the expression of the EGFR pathway and a decrease in the concentration of PI3K/Akt proteins in MCF-7 breast cancer cells and consequently inhibits the proliferation and survival of cancer cells [68]. In the study by Jamshidi-Adegani et al. [69], the synergistic effects of **U-IX** and **U-X** were assessed. **U-IX** is a β-boswellic acid derivative that exhibits stronger cytotoxic, antiproliferative, and antimetastatic effects against cancer cells, whereas **U-X** demonstrated less apoptosis induction in normal MCF-10A breast cells. Both derivatives increase the level of the proapoptotic Bax protein and additionally inhibit the proliferation of cancer cells by increasing the concentration of the p53 protein. Additionally, U-X exhibited chemo-preventive properties in the study. Therefore, the synergistic effects of the two derivatives, **U-IX** and **U-X**, seem plausible in anticancer treatment strategies by improving the bioavailability and pharmacodynamic properties of β-boswellic acid derivatives [69]. Mazzio et al. [70] investigated the mechanism of action of **U-X** in the MDA-MB-231 cell line. Their results demonstrated that **U-X** stimulates the expression of PERK (protein kinase RNA-like endoplasmic reticulum kinase) during the unfolded protein response (UPR), ultimately triggering programmed cell death. Additionally, **U-X** stimulates the expression of tumor suppressor genes (TSGs), which inhibits the mTOR pathway [70].

### 3.3. Asiatic Acid and Its Derivatives

**Figure 9:**
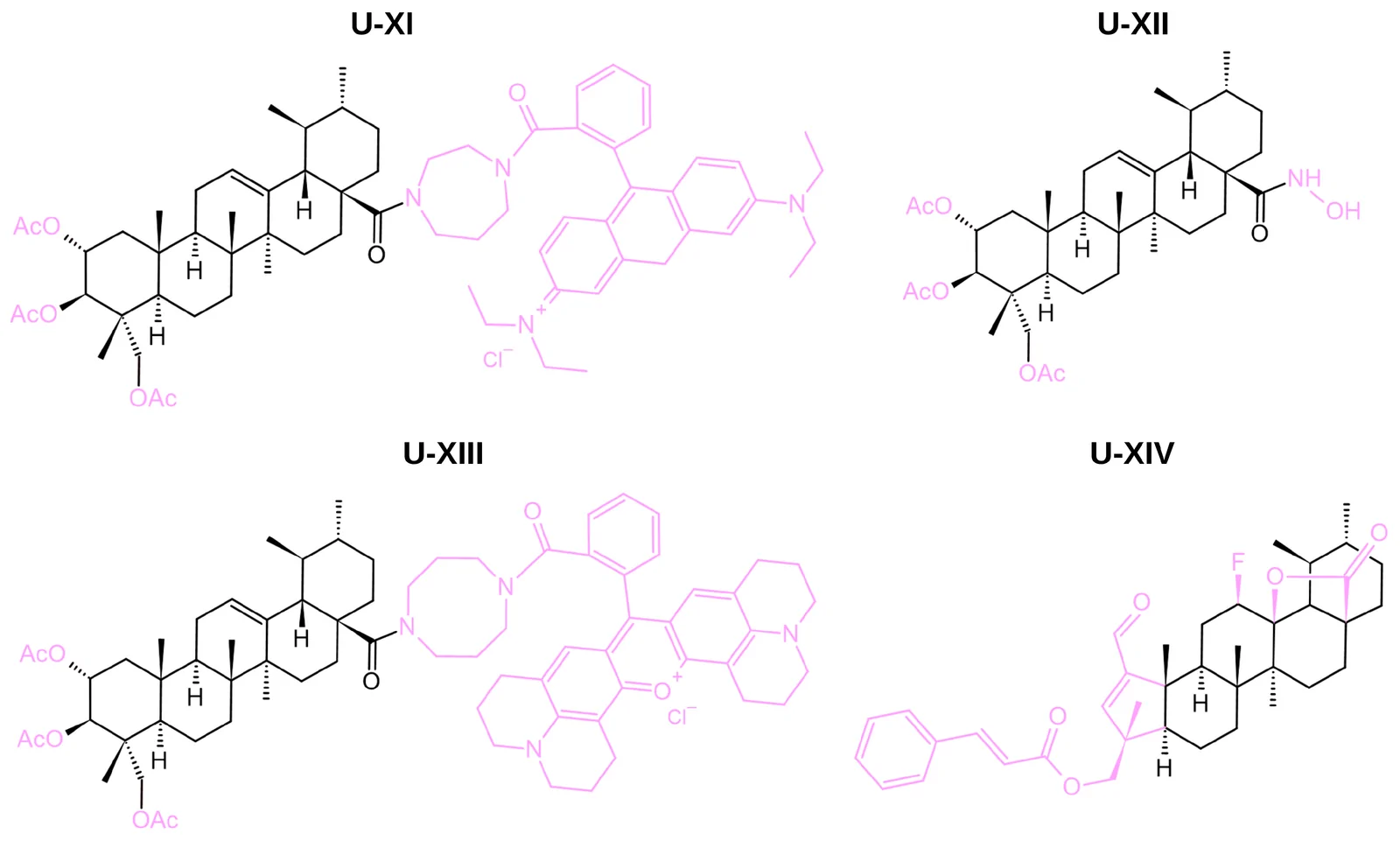
The structures of asiatic acid derivatives. Structural differences between the compounds are highlighted in pink.

Asiatic acid (ASA) is a natural acid from the pentacyclic triterpenoid family, naturally encountered and derived from the traditional medicinal herb *Centella asiatica* [80]. It has been widely used in traditional medicine for its anti-inflammatory, wound-healing, and hepatoprotective properties. Thus, ASA was investigated for its anticancer effects against breast cancer as well [81]. Studies on MCF-7 and MDA-MB-231 breast cancer cell lines have shown that asiatic acid demonstrates cytotoxic activity through different mechanisms depending on the cell line. In the MCF-7 cell line, Zhu et al. demonstrated that asiatic acid induces apoptosis by generating reactive oxygen species and reducing ATP content. Additionally, it activates transcription of the AMPK and NF-kB pathways, which reverses the DOX resistance, providing sensitizing effects as well [82]. Gou et al., in their studies on asiatic acid, showed that in the MDA-MB-231 cell line, the acid inhibited the PI3L/AKT signaling pathway, which is responsible for promoting cell growth, survival, and metabolism [83]. Kraft et al. [71] tested the cytotoxic effects of asiatic acid and its derivatives in six cell lines, including the MCF-7 breast cancer cells. The most cytotoxic derivative was the one conjugated with homopiperazinyl rhodamine B (**U-XI**), where the EC_50_ obtained by the SRB assay was 7.1 nM, which represented a significantly higher potency compared to native asiatic acid, where this value was over 30 µM. However, it was more active against the A375 line, which was selected for further studies (Figure 9) [71]. Gonçalves et al. synthesized 18 new asiatic acid derivatives. Compound **7**, with three acetoxy groups at C2, C3, and C23 and a hydroxylamine group at C-28 (**U-XII**), proved to be the most cytotoxic with an IC_50_ of 9.93 µM on the MCF-7 line (Figure 9) [72]. Heise et al. [73] also synthesized derivatives of asiatic acid conjugated with 1,5-diazacycloacetate and rhodamine B or rhodamine 101. The most active derivative against the tested breast cancer cell lines MCF-7, MDA-MB-231, HS5787 and T-47D was the derivative of asiatic acid with rhodamine 101 (**U-XIII**). The IC_50_ value obtained in the SRB assay was 0.6, 3.96, and 8.18 nM for the MDA-MB-231, MCF-7, and T-47D lines, respectively, after 96 h treatment. Additionally, the ability of the most cytotoxic compound to induce apoptosis was tested on the MDA-MB-231 cell line. It was demonstrated that cells treated with the compound at lower concentrations were inhibited in their proliferation and growth, while higher concentrations of the compound, around 250 nM and more, significantly induced apoptosis (Figure 9) [73]. Gonçalves’s team also synthesized new fluorinated derivatives of asiatic acid (**U-XIV**). The most cytotoxic compound with cinnamic ester as a substituent, at the C23-OH position in the MCF-7 cell line, reached an IC_50_ of 0.84 µM after 72 h of incubation in the MTT assay. However, the HT-29 colon cell line and the HeLa ovarian cancer cell line were selected for further studies on the mechanism of action as this compound was more active against them (Figure 9) [74].

### 3.4. Corosolic Acid

**Figure 10:**
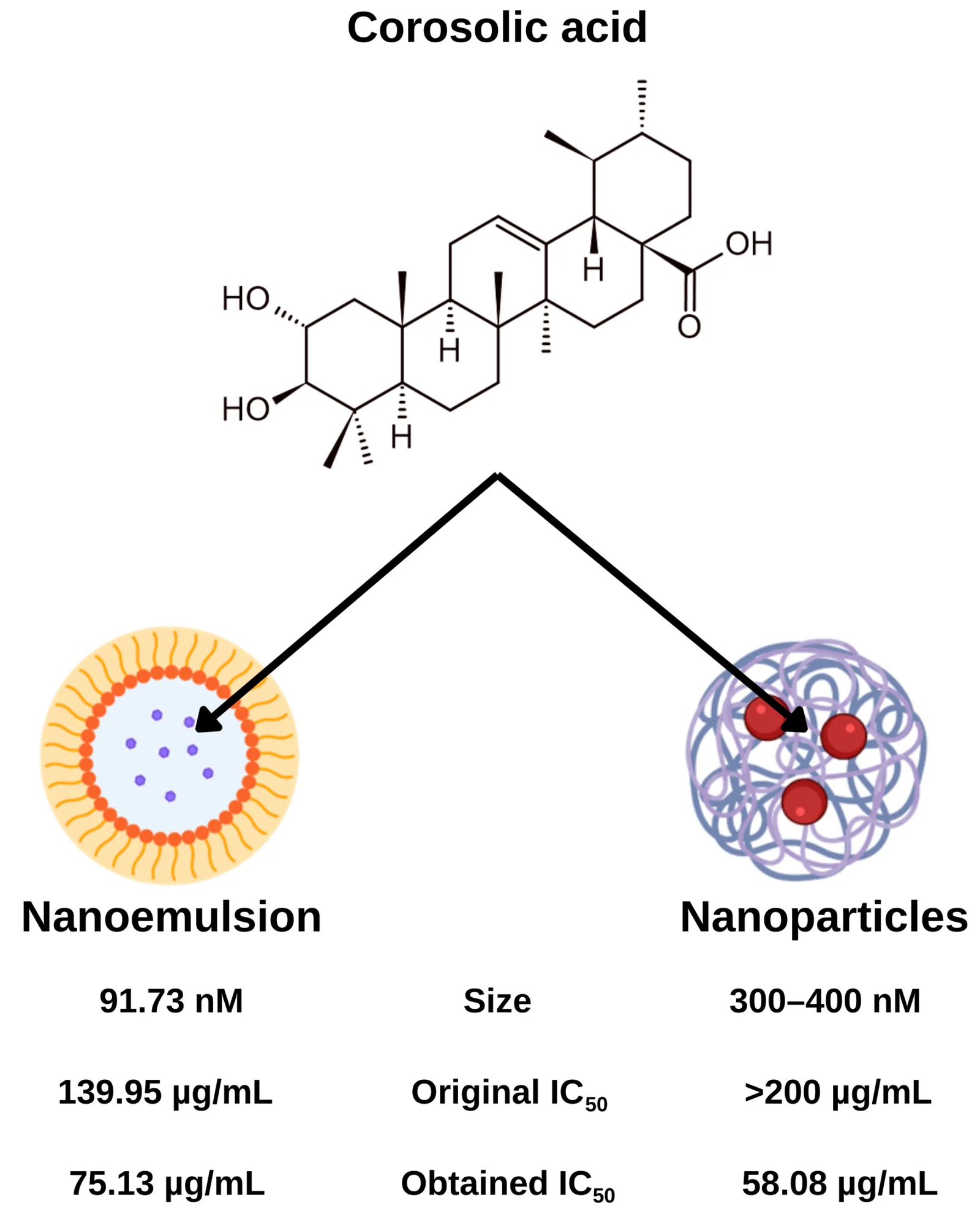
The comparison of obtained nanoemulsion and nanoparticles with the corosolic acid as an active compound. Purple dots and red spheres represent the active ingredient, corosolic acid.

Corosolic acid is another example of pentacyclic triterpenoid acids with the ursane skeletal type. Its structure, solubility, and synthetic availability are quite similar to maslinic acid due to them featuring two hydroxyl groups at the C2 and C3 atoms of the pentacyclic triterpenoid core. Corosolic acid is mainly isolated from the banana tree (*Lagerstroemia speciosa*). However, it is also present in other plants such as *Prunus armeniaca*, *Prunus padus* Linne, *Eucalyptus globulus* and *Chinese hawthorn*. CA displays a wide range of activities including hypoglycemic, anticancer, anti-inflammatory, antioxidant, and cardioprotective effects [84]. While corosolic acid is famously known for its antidiabetic properties, the compound has also been researched for its anticancer effects [85,86]. In an article by Son et al. [85], the authors researched the anticancer properties of corosolic acid against triple-negative breast cancer cell line (MDA-MB-231). CA reduced cell viability by up to 35% after 48 h of treatment at 10 µM concentration. Further analysis revealed that corosolic acid exhibits proapoptotic activity by elevating the levels of Bax and cleaving caspase 3 post-treatment. According to the authors, the origin of the anticancer properties of CA against MDA-MB-231 cells seems to be caused by the generation of ROS. Furthermore, this compound also substantially decreased the VEGF protein, affecting angiogenesis, and impaired the cells’ metastatic capability as was shown in the wound healing assay [85]. In a study by Jasim et al. [86], the authors tested corosolic acid not only against the triple-negative MDA-MB-231 breast cancer cell line but also against the hormone-receptor-positive MCF-7 cell line. Interestingly, corosolic acid was more potent against MDA-MB-231 rather than MCF-7 cells, with IC_50_ equal to 20.12 and 28.50 µM, respectively. Analysis of proapoptotic effects on both cell lines displayed that only MDA-MB-231 exhibited an increase in early and late apoptotic population of analyzed cells, whilst the apoptotic population of MCF-7 cells treated with CA remained similar to control. This shows that CA selectively demonstrates proapoptotic activity against triple-negative breast cancer cells. Utilizing real-time Polymerase Chain Reaction (PCR) and Western blotting, the authors provided further confirmation of proapoptotic effects of CA as it managed to notably increase the concentration of caspases 8, 9 and 3, which suggests that corosolic acid induces both pathways of apoptosis. Finally, corosolic acid significantly decreased the levels of phosphorylated janus kinase (JAK2) and STAT3 proteins without affecting the inactivated JAK2 and STAT3 proteins. This provides a direct confirmation of corosolic acid’s capability to block the phosphorylation of those proteins, decreasing the survivability of MDA-MB-231 cells [86]. Nonetheless, the main issue of corosolic acid continues to be its poor aqueous solubility, making it impractical as a potential anticancer drug candidate. One of the solutions to such a problem is utilizing nanoscale delivery systems to combat this issue [87,88]. In an article by Petchimuthu et al. [87], the authors developed a biodegradable delivery system for corosolic acid utilizing kafirin, a highly hydrophobic protein isolated from red sorghum grains. Utilizing coacervation methodology with glutaraldehyde as a cross-linking agent and Tween 80 as a stabilizer, the authors obtained nanoparticles 300 to 400 nm in diameter with 81.13% of encapsulation efficiency. The research into anticancer effects of the obtained nanoparticles (CA-Kaf) revealed that CA-Kaf displayed IC_50_ equal to 58.08 µg/mL, a notably higher potency in comparison to original CA with IC_50_ equal to over 200 µg/mL. Fluorescent imaging with AO/EB (Acridine Orange/Ethidium Bromide) and DAPI (4′,6-diamidino-2-phenylindole) showed morphological changes typical of apoptosis, such as cytoplasm shrinkage, membrane blebbing, and severe nuclear fragmentation. Similarly to original CA, the obtained nanoparticles also elevated ROS levels, which decreased the mitochondrial membrane potential [87]. In contrast to kafirin-based nanoparticles, Gaur et al. [88] developed lipid-based nanoemulsion (CA-NE) with a significantly lower droplet size equal to 91.73 nm. The authors applied lemongrass oil, a non-ionic surfactant Cremophor EL, and co-surfactant Transcutol HP to create a stable monodispersed system with high initial drug content equal to 98%. The authors tested obtained a product against MCF-7 cells and compared it to the original CA. MTT assay revealed that the created nanoemulsion indicated two-fold reduction in IC_50_ concentration with 75.13 µg/mL for CA-NE and 139.95 µg/mL for original CA [88]. Despite that, the authors did not perform a detailed analysis of the mechanism of anticancer effects of the newly obtained system, nor was the safety profile of this nanoemulsion investigated (Figure 10).

## 4. Lupane Skeletal Type

In contrast to other pentacyclic triterpenoids which feature five six-membered rings connected to each other, the core of the lupanes has one five-membered ring (ring E). Furthermore, betulinic acid, the main representative acid with the lupane skeletal type, also has an isopropenyl group at the C19 position instead of an endocyclic double bond between C12 and C13. Those structural changes negatively impact the aqueous solubility of betulinic acid, making them highly lipophilic. It also affects the chemical modification of betulinic acid. While the majority of reactions still focus on the hydroxyl group at the C3 position or the carboxylic group at C17 in a similar way to oleananes and ursanes, some unique to lupanes reactions have been utilized as well [14]. For instance, the exocyclic double bond can be converted to ketone via Lemieux–Johnson oxidation utilizing OsO_4_/NaIO_4_ reagents [89] or allylic radical bromination followed by nucleophilic substitution at C30 [90]. Promising derivatives of betulinic acid with anticancer properties against breast cancer are gathered in Table 3.

**Figure 11:**
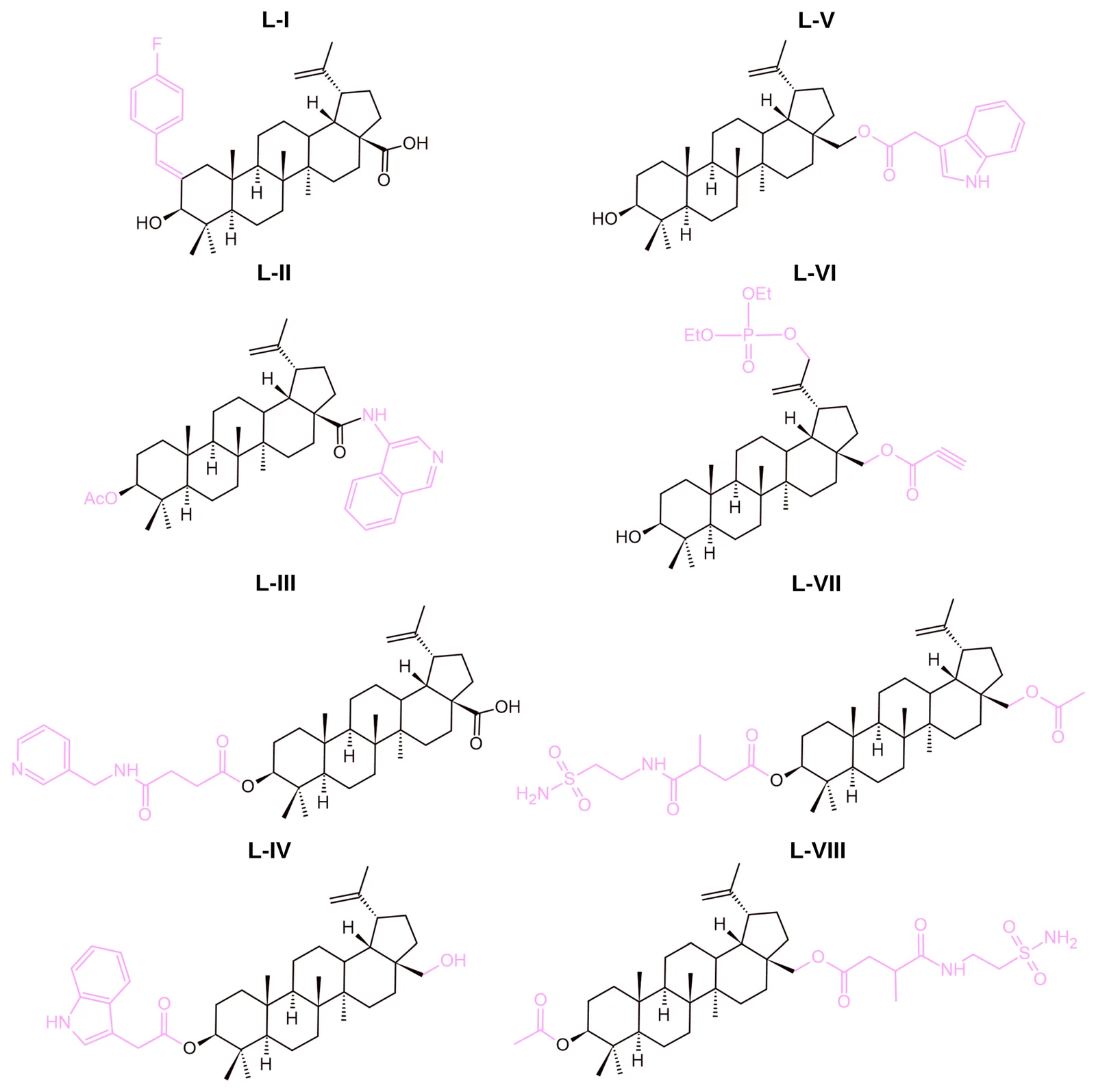
The structures of derivatives with lupane skeletal type. Structural differences between the compounds are highlighted in pink.

Betulinic acid demonstrates a variety of anticancer activities in the treatment of breast cancer. The main mechanism behind its antitumor effects is related to its capability to decrease mitochondrial membrane potential, activate kinases p38 and JNK, and increase the ROS generation, which often correlates with the mitochondrial pathway of apoptosis [98,99,100]. There are also reports of betulinic acid inhibiting aerobic glycolysis by targeting GRP78 protein and caveolin-1/NF-κB/c-Myc pathway, which essentially starves the tumor [101,102]. Those features make betulinic acid of significant interest for development of novel anticancer compounds. In the last decade, a number of betulinic acid derivatives have been synthesized and researched for their anticancer effects (Figure 11) [91,92,93,94,95,96,97].

It should be noted that not every work has provided an in-depth analysis of anticancer properties of the respected derivatives of betulinic acid [91,92,93]. For instance, in the work by Gupta et al., the authors synthesized a batch of novel betulinic acid derivatives with various benzylidene fragments attached to C2 position of the core structure [91]. In their study, the authors tested their new structures against a number of cancer cell lines such as A-549 (lung), PC-3 (prostate), HCT116 (colon), MIA PaCa-2 (pancreatic) and MCF-7 (breast) and normal human fibroblasts. The most active compound against MCF-7 breast cancer cells was **L-I** with a 4-fluorobenzylidene substituent, which displayed an IC_50_ equal to 1.18 µM and SI equal to 12.22. Considering its potent cytotoxic effect and mildly promising safety profile, this compound is worth investigating in the future against breast cancer [91]. In other work by Hoenke et al., the authors presented a new series of betulinic acid derivatives, among which the 4-isoquinolinyl amide of 3-O-acetyl-betulinic acid (**L-II**) was the most promising structure targeting breast cancer MCF-7 cells, with EC_50_ equal to 4.65 ± 0.5 µM and SI equal to 29.0 [92]. Nonetheless, the **L-II** compound was more active against melanoma A375 cells, which were chosen for further analysis of anticancer properties with apoptosis, cell cycle and microscopy evaluations performed post-treatment. Considering the favorable initial results, a more detailed evaluation of the mechanism of anticancer activity of this compound should be performed in the future [92]. Bildziukevich et al. synthesized a series of picolyl amides of betulinic acid, which were tested against leukemia (CEM), melanoma (G-361), cervical (HeLa) and breast (MCF-7) cancer cells [93]. Similarly to previous work, melanoma cells were the most affected by newly synthesized compounds, especially by **L-III**, a structure featuring a 3-picolylamine connected to the C3-oxygen of the lupane core via a succinate linker. Thus, other methods of research, including assessment of apoptosis, cell cycle and numerous apoptosis-related proteins (PARP-1, caspase-7, AKT and MAPK), were focused on melanoma cells. Nonetheless, the IC_50_ value of **L-III** against MCF-7 (1.4 ± 0.1 µM) and the SI value (35.71) provide a promising cytotoxicity and safety profile for more in-depth research of the effects of this compound on breast cancer [93]. Among the aforementioned articles, two reported the anticancer activity of novel betulin derivatives against breast cancer with an introduced indole fragment, albeit at different locations. In an article by Rzepka et al., the change was made at C3 (**L-IV**), while in the article by Bębenek et al., modification was performed at C3 (**L-V**) [94,95]. In both cases, the MCF-7 cell line was the most prone to the activity of the respective compounds, with EC_50_ values equal to 67 µM and 35 µM accordingly. However, a difference in mechanism of activity was noted for those articles. For instance, **L-IV** displayed more potent cytotoxic activity via DNA damage, while **L-V** revealed cytostatic effects without fragmenting DNA. In the article by Orchel et al., the authors demonstrated five new derivatives with introduced diethoxyphosphoryl and/or acetylenic groups at main connecting points of the lupane core, namely C3, C28 and C30 [96]. Among them, the most active compound, **L-VI**, featured the diethoxyphosphoryl ester at the C30 atom and the acetylenic group at the C28 atom of the lupane core. The compound indicated potent cytotoxic activity with IC_50_ values equal to 5.34 ± 0.03 and 2.12 ± 0.59 µM for MCF-7 and SK-BR-3, respectively. This addition was directly targeting the main issue of betulinic acid, namely its physicochemical properties, especially its poor aqueous solubility. **L-VI** caused notable damage to mitochondrial membrane via the decrease in mitochondrial membrane potential. Assessment of ROS generation revealed a unique pattern, where compound **L-VI** induced ROS generation in MCF-7 cells but reduced it in SK-BR-3 cells. As stated by the authors, such fluctuations in ROS levels can contribute to the revealed damage to mitochondrial membrane, inducing cell death. Another analysis displayed that compound **L-VI** also increased caspase 3 levels, an important proapoptotic signal. Nonetheless, the morphological analysis revealed a more necrotic-like type of cell death rather than apoptosis, despite the confirmed signals of apoptosis. Considering those inconsistencies, more in-depth research of compound activity is required. Lastly, Güttler et al. described a small number of new compounds featuring carbonic anhydrase inhibition as the main source of anticancer activity of their new derivatives [97]. Similarly to other analyzed studies, the main structural modification was performed at the C3 and C28 atoms with the addition of acetyl and sulfonamide groups. Compound **L-VII** featured an acetyl group at the C28 atom and a sulfonamide group at the C3 position of the lupane moiety, while compound **L-VIII** had those fragments reversed. The sulfonamide group is an established CA inhibitory group; thus, its introductions to the core structure of betulin is necessary to facilitate CA inhibition effects [103]. Both structures and their precursors were analyzed against a panel of breast cancer cell lines (HS578T, MDA-MB-231, BT-20, MCF-7, T47D, SK-BR-3). The three most affected cell lines were HS578T, MDA-MB-231 and MCF-7. Interestingly, MCF-7 cells were affected even by precursors, while HS578T and MDA-MB-231 displayed certain levels of resistance towards them by being actively inhibited only by **L-VII** and **L-VIII**. Further assessment of anticancer effects of **L-VII** and **L-VIII** revealed that those structures increased the apoptotic cell population post-treatment, particularly in MDA-MB-231 cells, where **L-VII** displayed 40% of apoptotic cells and 5% of necrotic cells and **L-VIII** exhibited 50% of apoptotic and 26% of necrotic cells. The effects of those compounds on proteins levels of CA IX and CA XII were studied in all three tested cell lines. CA IX levels were not much affected by **L-VII** or **L-VIII** in HS578T cells, while CA XII levels were not affected by MCF-7 cells. In contrast, MDA-MB-231 cells showed notable decrease in both CA IX and CA XII post-treatment with **L-VII** and **L-VIII**. However, new derivatives managed to reduce CA XII protein expression in HS578T cells and CA IX in MCF-7 cells; especially visible was the effect of **L-VIII**. Thus, both compounds revealed promising results against numerous breast cancer cell lines as potent carbonic anhydrase inhibitors, especially against MDA-MB-231 cell line. Those compounds should be further researched in the future, utilizing in vivo models to confirm the activity and safety of new structures.

## 5. Discussion and Conclusions

Pentacyclic triterpenoid acids are well-known for their anti-inflammatory, antioxidant, antibacterial, and antiviral properties. Successfully used for many years in various fields of medicine, they have also attracted the interest of scientists in the fight against cancer. Interesting observations were made for the mechanisms of anticancer activity for natural pentacyclic triterpenoid acids. While the majority of them caused the death of cancer cells via a multi-targeting approach with numerous mechanisms of anticancer activity, nearly all of those structures displayed the ability to generate ROS. Such activity was noted for oleanolic, glycyrrhetinic, arjunolic, β-boswellic acid, asiatic, and corosolic and betulinic acids. Surprisingly, maslinic acid exhibited the opposite effect and reduced the ROS levels, while its mechanism of anticancer activity was ER-dependent. Cell cycle arrest was induced via regulation of the MAPK/ERK pathway. Pentacyclic triterpenoids not only affect cancer cells, modifying their proliferation, differentiation, and metabolism, but also inhibit metastatic properties by reducing cell migration and adhesion. Nonetheless, pentacyclic triterpenoid acids notably have certain disadvantages. For instance, their bioavailability is poor due to their high lipophilicity, caused by the bulky pentacyclic triterpenoid core, which reduces their aqueous solubility. Another weakness of the natural pentacyclic triterpenoid acids is their overall high IC_50_ concentrations against breast cancer, often showcasing values over 20 µM. Such substantive numbers can become a significant issue in current drug development, where compounds with large IC_50_ concentrations often fail in transition to in vivo research and fail to pass the early stages of clinical trials [104,105].

Taking into consideration these limitations, numerous authors targeted not only the solubility issues but also searched for structural motifs, which would increase the potency of those compounds or introduce new mechanisms of anticancer activity. In previous years, the most researched structural type was oleanane, with 43.59% of analyzed manuscripts showcasing new derivatives with this skeletal type. In contrast, ursanes and lupanes were less popular, with the aforementioned manuscripts noting 35.90% and 20.51% of showcased new derivatives, respectively. The most popular locations for synthetic modifications of all-natural pentacyclic triterpenoid acids were atoms that featured hydroxyl and carboxylic groups, as those structural fragments are more reactive than the pentacyclic triterpenoid core. Examples of compounds with newly introduced fragments attached to carbon of the carboxylic group are **O-II**, **O-III**, **O-VII**, **O-VIII**, and **O-XI**. In the case of **O-IV**, methyl ester was attached to the C28 position; in **O-V**, the imidazolide group was added, and for the **U-III** compound, a 1-(2-amino-ethylamino) substituent was introduced. Another group of compounds comprises modified derivatives introduced at the carbon atom with the attached hydroxylic group, mainly at C3, such as **L-III**, **L-IV** and **L-V**. In the **O-VI** compound, cinnamoyl and benzyl ester moieties were attached. To obtain **O-XVI**, the 3-hydroxyl group was transformed with lipophilic fragments or different amino acids such as L-selenomethionine and L-methionine. For **U-I** in the C3 position, the nitrosoacetyl group replaced the hydroxyl group, and in **U-II**, the analog 3,4,5-methoxybenzoyl group was introduced. Furthermore, several compounds’ modifications were made at both carbons with hydroxylic and carboxylic groups. For the **O-XVII** compound, the hydroxyl group was substituted with the ketone group at the C3 position, and in the C28 position, the carboxyl group was converted into the methoxycarbonyl group. To obtain the **U-VI** compound, the acetyl group was attached at C3 and 2-amino-1,3,4-oxadiazole at C28. In the case of **U-VIII**, the piperazine group was introduced at C3 and a carbomethoxy group at C28. Regarding **O-XII** synthesis, hydroxyl groups in positions C2 and C3 were protected and urea moieties were incorporated at the C-28 position. In **L-VII**, the acetyl group was added at C28 and sulfonamide group at C3; for **L-VIII**, these groups were reversed with the sulfonamide group at C28 and acetyl group at C3. Another structural change was performed at the C30 position during **O-XIV** and **O-XV** synthesis, where tetraglycine fragments were introduced. To obtain **O-XIII**, modifications in both C3 and C30 were performed where the acetyl group and methyl ester group were substituted, respectively. The remaining compounds were acquired mostly after modifications at the C2, C4 and C5 positions.

From the assessment of numerous studies in previous years, certain patterns were noted. For instance, some of the manuscripts demonstrated only the synthesis and cytotoxicity assessments of their new compounds without analyzing the mechanism of their anticancer effects. Another issue is better activity against other types of cancer. Some of the works featured the cytotoxicity assessment against breast cancer, but due to better performance against other cancer cell lines, breast cancer models were not selected for detailed mechanistic follow-up of anticancer effects of novel compounds. In such cases, a detailed analysis of the mechanism of the anticancer activity against breast cancer cell lines is required before deriving conclusions since the mechanism can vary greatly depending on the properties of the cell line.

Nonetheless, the main issue of pentacyclic triterpenoid acids, their poor bioavailability, remains unsolved. Among various ways to improve the solubility of the natural pentacyclic triterpenoid acid of interest is to utilize nano-delivery systems such as nanoemulsions or nanoparticles. Unique comparison was noted for corosolic acid, with two works showcasing those different nano-delivery systems. However, despite the authors’ successful formulation of nanoemulsions and nanoparticles of corosolic acid, their IC_50_ values were still on the higher side with IC_50_ over 50 µg/mL. This issue can potentially be attributed to the majority of the natural pentacyclic triterpenoid acid due to the original large concentrations required for anticancer activity. However, taking into account the novel synthetic derivatives with significantly lower IC_50_ concentrations against breast cancer, exploring the nano-delivery systems for those derivatives can be a worthwhile research topic to target both the solubility and the potency issues of original pentacyclic triterpenoid acids.

In conclusion, the pentacyclic triterpenoid acids provide a promising core for the synthesis and research of novel anticancer compounds targeting breast cancer. The works from previous years indicated new derivatives with increasingly stronger anticancer effects against breast cancer. However, the main issues of the natural pentacyclic triterpenoid acids, mainly their bioavailability and poor aqueous solubility, remain unresolved. Future research into synthesis of novel pentacyclic triterpenoid acids will require the improvement of not only the cytotoxicity but also the bioavailability of the natural pentacyclic triterpenoid acids to provide safe and potent cytotoxic drugs.

## Figures and Tables

**Table 1:** Synthetic derivatives of pentacyclic triterpenoid acids with oleanane skeletal type and their anticancer activity against breast cancer. O—Oleanane, nm—not measured.

Name	Breast Cancer Cell Lines	IC_50_ in µM	Antiproliferative Activity	Refs.
**O-I**	MCF-7	9.8	ROS generation, apoptosis induction, cell migration inhibition	[21]
	MDA-MB-231	11.2		
**O-II**	MCF7/ER+	3.2	Autophagy induction, cell cycle arrest, cell migration inhibition, apoptosis induction	[22,23]
	MCF7/ER−	1.63		
	MDA-MB-231/EGFR+	21		
	MDA-MB-231/EGFR−	1.72		
	SK-BR-3	3.89		
**O-III**	MCF7/ER+	6.58	Autophagy induction, cell cycle arrest, cell migration inhibition, apoptosis induction	[22,23]
	MCF7/ER−	3.53		
	MDA-MB-231/EGFR+	>50		
	MDA-MB-231/EGFR−	3.4		
	SK-BR-3	3.87		
**O-IV**	MDA-MB-231,	nm	ROS generation, GSH depletion, SOD2 inhibition, Wnt/β-catenin pathway inhibition, MAPK/Akt inhibition	[24,25,26,27]
	MDA-MB-468	nm		
**O-V**	SUM159 MDA-MB-231	nm	Apoptosis induction, cell cycle arrest	[28]
**O-VI**	MCF-7	1.79	-	[29]
**O-VII**	MCF-7	7.48 ± 0.33	-	[30]
**O-VIII**	MCF-7	7.61 ± 0.33	-	[30]
**O-IX**	EMT-6	nm	-	[31]
**O-X**	EMT-6	nm	-	[31]
**O-XI**	MCF-7	15.71 ± 1.86	-	[32]
	MDA-MB-231	17.98 ± 1.00		
**O-XII**	MCF-7	2.8 ± 1.8	-	[33]
**O-XIII**	MCF-7	4.5 ± 0.1	Apoptosis induction, cell cycle arrest	[34]
**O-XIV**	MCF-7	5.0 ± 0.3	Apoptosis induction, β-tubulin polymerization inhibition	[35]
**O-XV**	MCF-7	6.20 ± 0.25	Apoptosis induction, DNA damage, β-tubulin polymerization inhibition	[36]
**O-XVI**	MCF-7	1.88 ± 0.20	-	[37]
	MDA-MB-231	1.37 ± 0.18		
**O-XVII**	MCF-7	20.7 ± 2.7	p-gp downregulation	[38]
	MCF-7/DOX	31.1 ± 4.0		

**Table 2:** Synthetic derivatives of pentacyclic triterpenoid acids with ursane skeletal type and their anticancer activity against breast cancer. U—ursane, nm—not measured.

Name	Breast Cancer Cell Lines	IC_50_ in µM	Antiproliferative Activity	Refs.
**U-I**	MCF-7	3.43	-	[60]
**U-II**	MCF-7	22.27	-	[61]
**U-III**	MCF-7	8.45	-	[30]
**U-IV**	MCF-7 MDA-MB-231	14.4 1.4	Change of cellular phenotype, arrest of breast cancer cells in mitosis	[62]
**U-V**	MCF-7	1.59	Apoptosis induction	[63]
**U-VI**	MCF-7	8.62 ± 2.33	-	[64]
**U-VII**	MCF-7	<0.1 ± 0.09	-	[65]
**U-VIII**	MCF-7	10.72	-	[66]
**U-IX**	MCF-7	nm	ROS generation, cell cycle arrest in G1 phase, apoptosis induction, EGFR/PI3K/Akt inhibition	[67,68]
**U-X**	MCF-7	nm	Increase in Bax and p53 protein levels, inhibition of mTOR pathway, apoptosis induction	[69,70]
	MDA-MB-231			
**U-XI**	MCF-7	0.0071	Apoptosis induction	[71]
**U-XII**	MCF-7	9.93	-	[72]
**U-XIII**	MDA-MB-231	0.00060	Apoptosis induction	[73]
	MCF-7	0.00396		
	T47D	0.00818		
	HS578T	0.1258		
**U-XIV**	MCF-7	0.84	-	[74]

**Table 3:** Synthetic derivatives of pentacyclic triterpenoid acids with lupane skeletal type and their anticancer activity against breast cancer. L—lupane; nm—not measured.

Name	Breast Cancer Cell Lines	IC_50_ in µM	Antiproliferative Activity	Ref.
**L-I**	MCF-7	1.18	-	[91]
**L-II**	MCF-7	4.65 ± 0.5	-	[92]
**L-III**	MCF-7	1.4 ± 0.1	-	[93]
**L-IV**	MCF-7	67	DNA damage	[94]
	MDA-MB-231	nm		
**L-V**	MCF-7	35	nm	[95]
	MDA-MB-231	nm		
**L-VI**	MCF-7	5.34 ± 0.03	ROS levels fluctuations	[96]
	SK-BR-3	2.12 ± 0.59		
**L-VII**	HS578T	In range of 8–14	CA IX and CA XII inhibition	[97]
	MDA-MB-231			
	BT-20			
	MCF-7			
	T47D			
	SK-BR-3			
**L-VIII**	HS578T	In range of 8–14	CA IX and CA XII inhibition	[97]
	MDA-MB-231			
	BT-20			
	MCF-7			
	T47D			
	SK-BR-3			

## Data Availability

No new data were created or analyzed in this study. Data sharing is not applicable to this article.

## Abbreviations

The following abbreviations are used in this manuscript:

18β-GA	18β-glycyrrhetinic acid
AA	Arjunolic Acid
AKT	Protein Kinase B
AMPK	5′AMP-activated Protein Kinase
AO/EB	Acridine Orange/Ethidium Bromide
ASA	Asiatic Acid
BA	β-boswellic Acid
Bax	Bcl-2 Associated X-protein
Bcl-2	B-cell lymphoma 2
CA	Corosolic Acid
CA-NE	Lipid-based Nanoemulsion
CDK-2	Cyclin-dependent kinase 2
DAPI	4′,6-diamidino-2-phenylindole
DMSO	Dimethyl sulfoxide
DOX	Doxorubicin
DVL	Dishevelled protein
EA	Echinocystic acid
EGFR	Epidermal Growth Factor Receptor
ELISA	Enzyme-Linked Immunosorbent Assay
ER	Estrogen Receptor
FAK	Focal Adhesion Kinase
FDA	Food and drug administration
GA	18β-glycyrrhetinic acid
GL	Glycyrrhizic Acid
GSH	Intracellular Glutathione Levels
HER-2	Human epidermal growth factor type 2
IL-10	Interleukin-10
IL-12	Interleukin-12
JAK2	Janus kinase 2
JNK	c-Jun N-terminal kinase
MA	Maslinic Acid
MAPK	Mitogen-Activated Protein Kinase
MDC	Monodansylcadaverine
MMP	Mitochondrial membrane potential
MMP-2	Metalloproteinase-2
MMTV	Mammary Tumor Virus
Nrf-2	Nuclear Factor Erythroid 2-related Factor 2
OA	Oleanolic Acid
PARP	Poly(ADP-ribose) polymerase
PCR	Polymerase Chain Reaction
PDGFR-α	Platelet-derived growth factor receptor-α
PERK	Protein Kinase RNA-like Endoplasmic Reticulum Kinase
PI3K	Phosphoinositide 3-kinase
PKM2	Pyruvate kinase muscle isozyme 2
ROS	Reactive Oxygen Species
SRB	Sulforhodamine B
STAT3	Signal Transducer and Activator of Transcription 3
TAM	Tumor-Associated Macrophages
TGF-β	Transforming growth factor beta
TNF-α	Tumor Necrosis Factor alpha
TSG	Tumor Suppressor Genes
UA	Ursolic Acid
UPR	Unfolded Protein Response
VEGF	Vascular Endothelial Growth Factor
